# Supplementary material for: Technology configurations for decarbonizing residential heat supply through district heating and implications for the electricity network
Source: iScience. 2026 Jul 9;29(7):116711. doi: 10.1016/j.isci.2026.116711 (PMC13378377; doi:10.1016/j.isci.2026.116711)
Supplement: Document S1. Figures S1–S41 and Table S1 [file mmc1.pdf]

iScience, Volume *29*

Supplemental information

Technology configurations for decarbonizing residential heat supply through district heating and implications for the electricity network

Christian Doh Dinga, Francesco Lombardi, Roald Arkesteijn, Arjan van Voorden, Sander van Rijn, Laurens J. de Vries, and Milos Cvetkovic

SUPPLEMENTAL INFORMATION

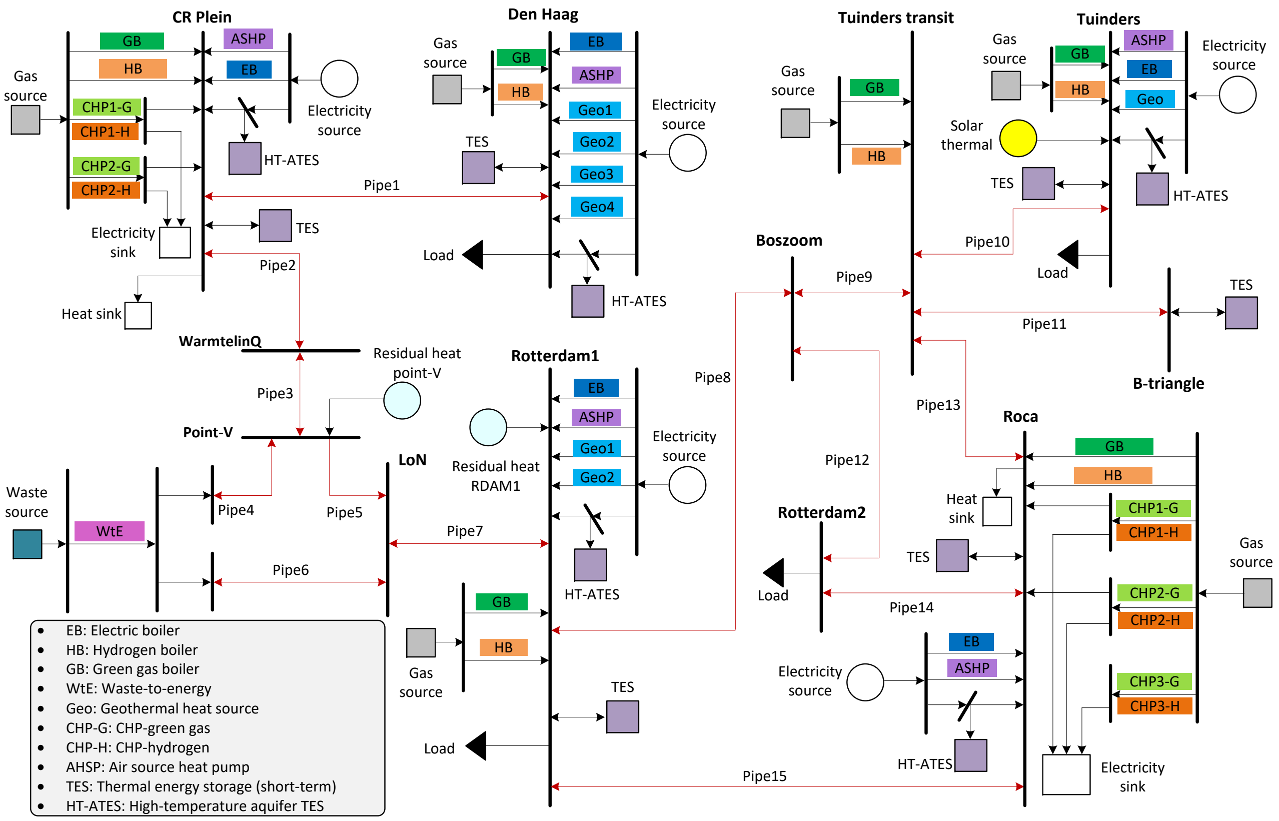

Figure S1. District heating network

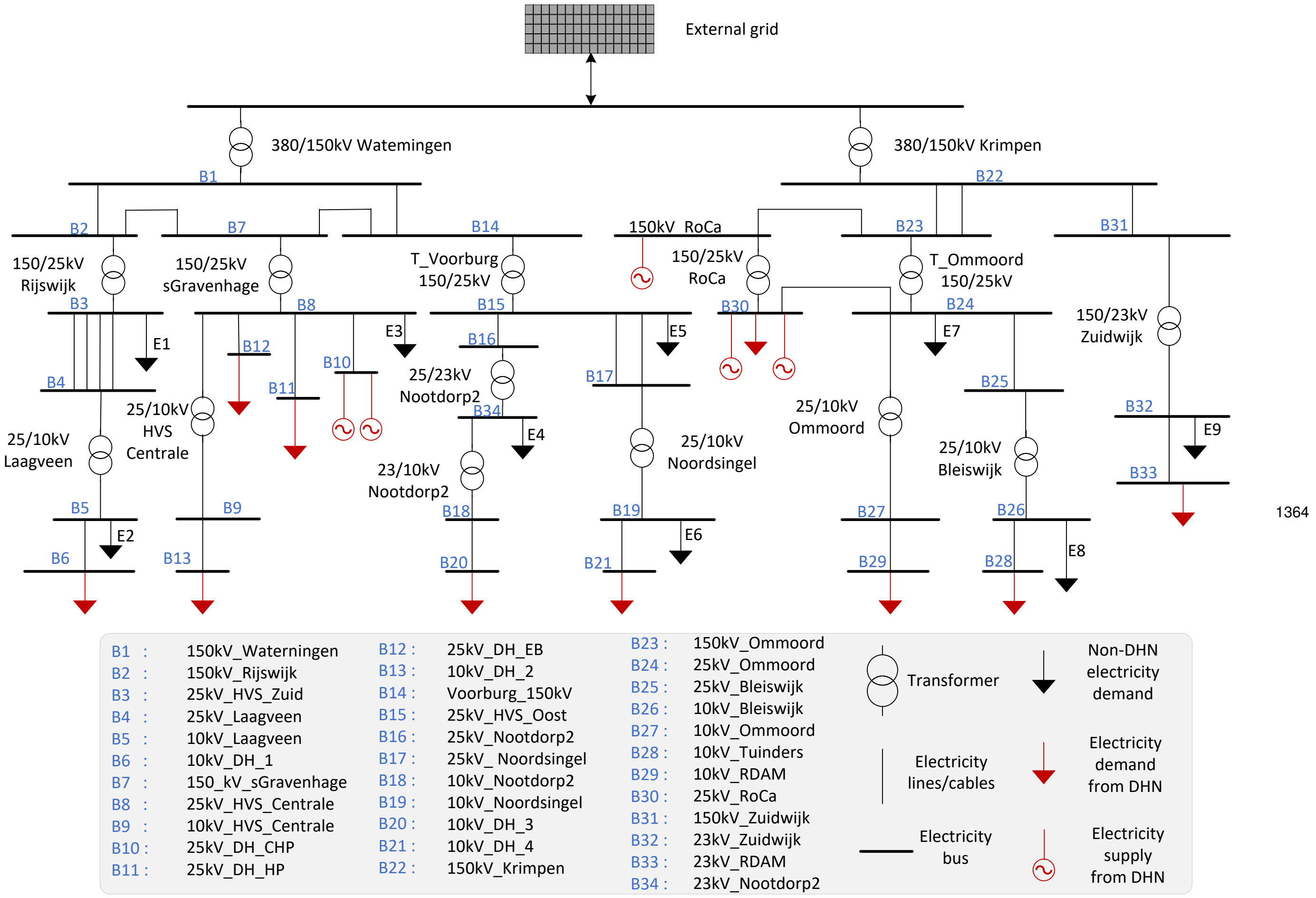

Figure S2. Electricity network

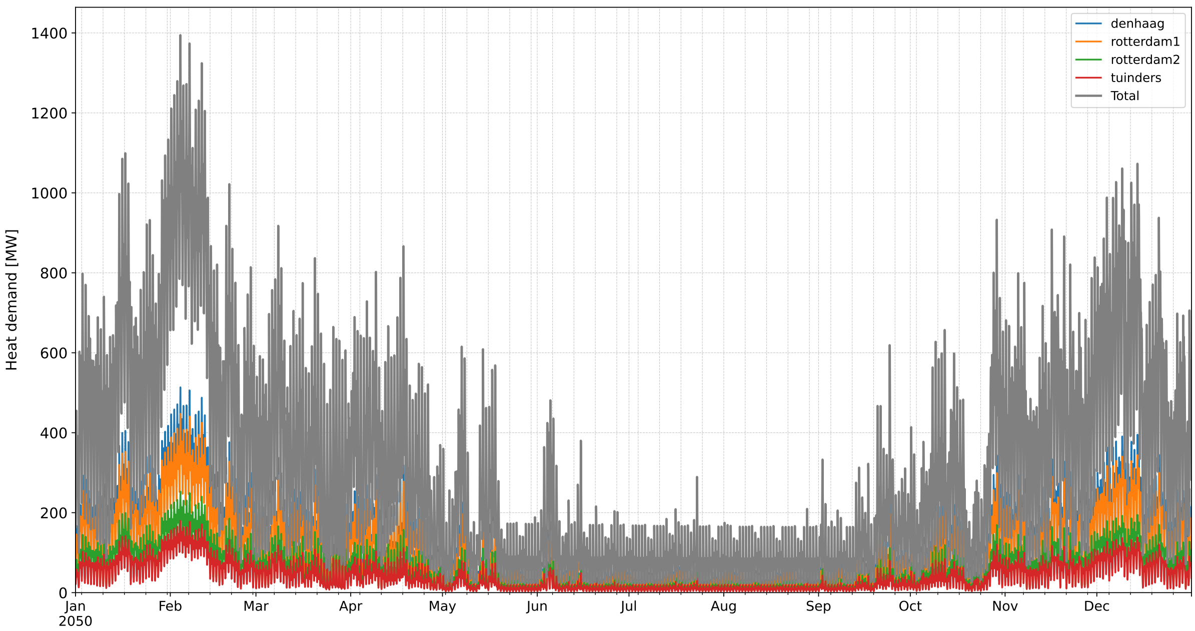

Figure S3. Projected heat demand by 2050

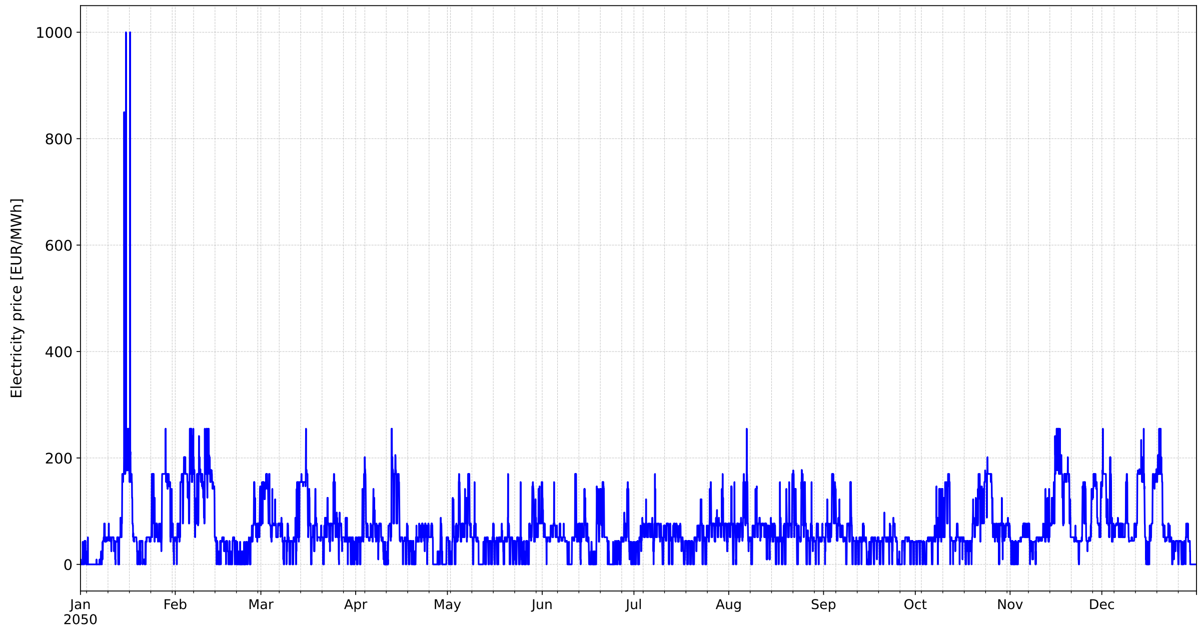

Figure S4. Electricity price

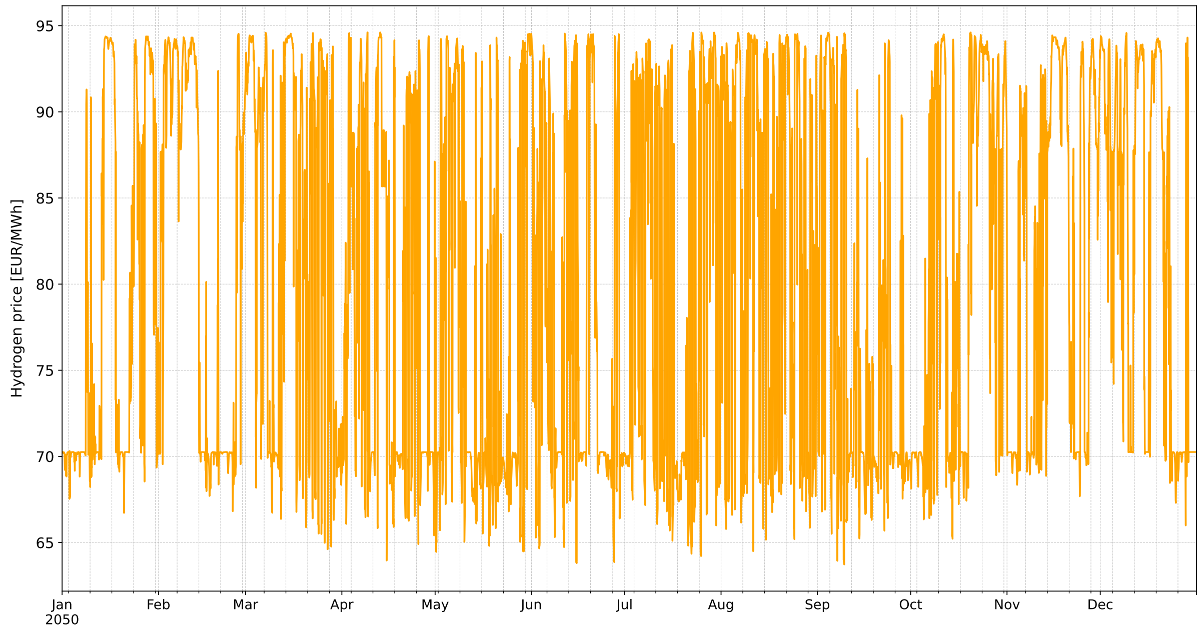

Figure S5. Hydrogen price

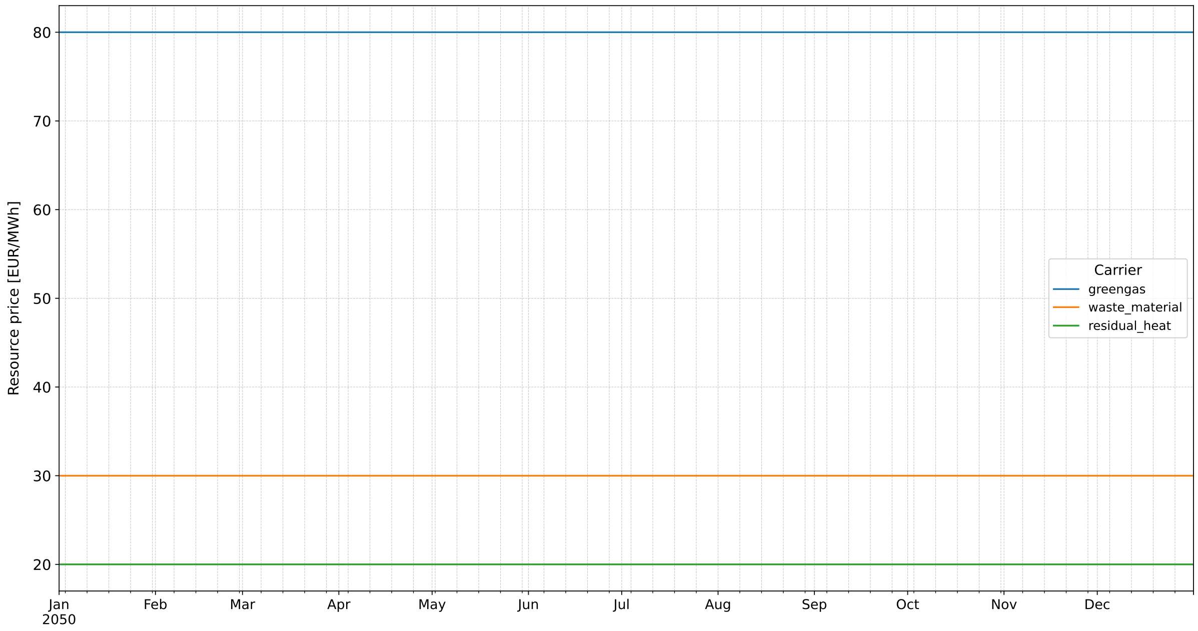

Figure S6. Green gas, waste material, and residual heat price

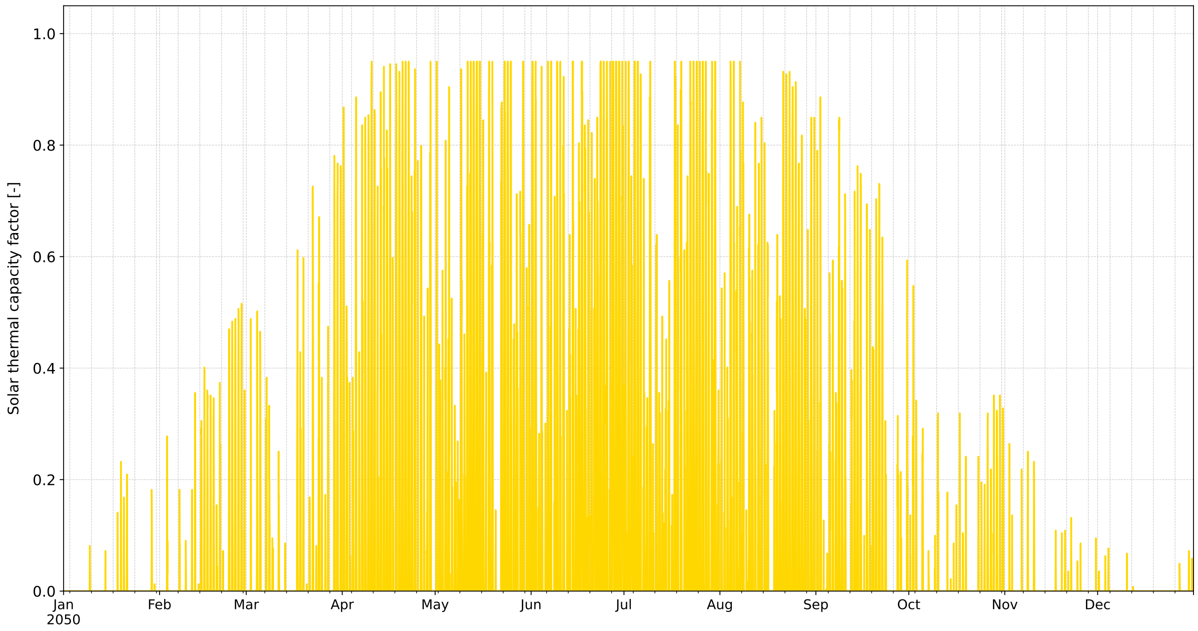

Figure S7. Solar thermal capacity factor

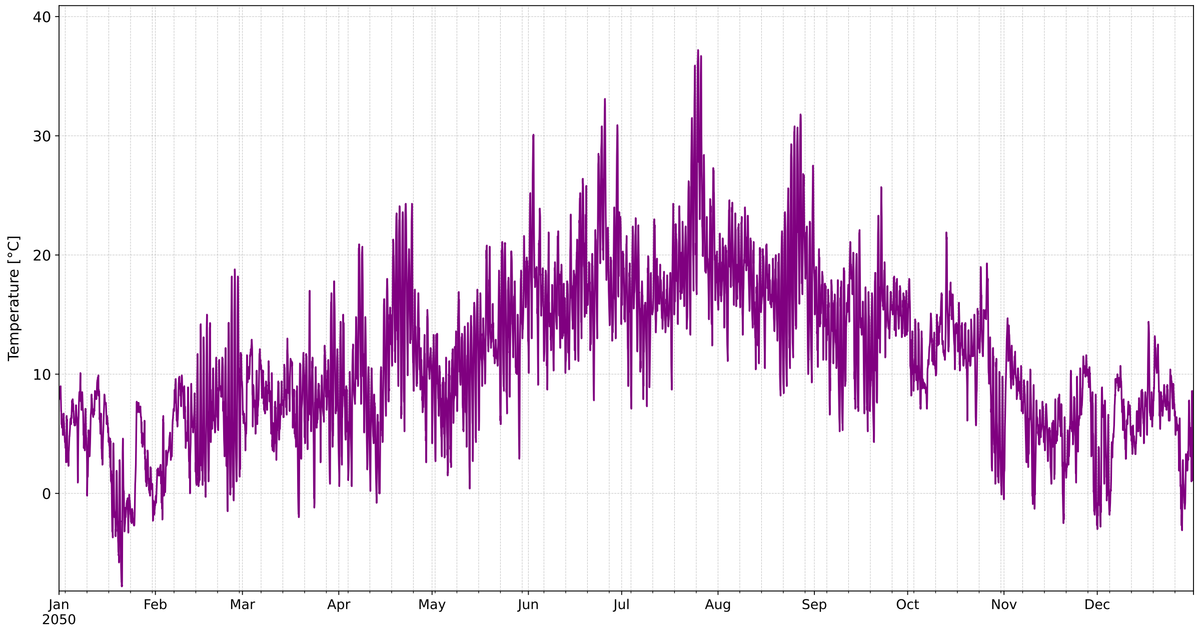

Figure S8. Ambient temperature

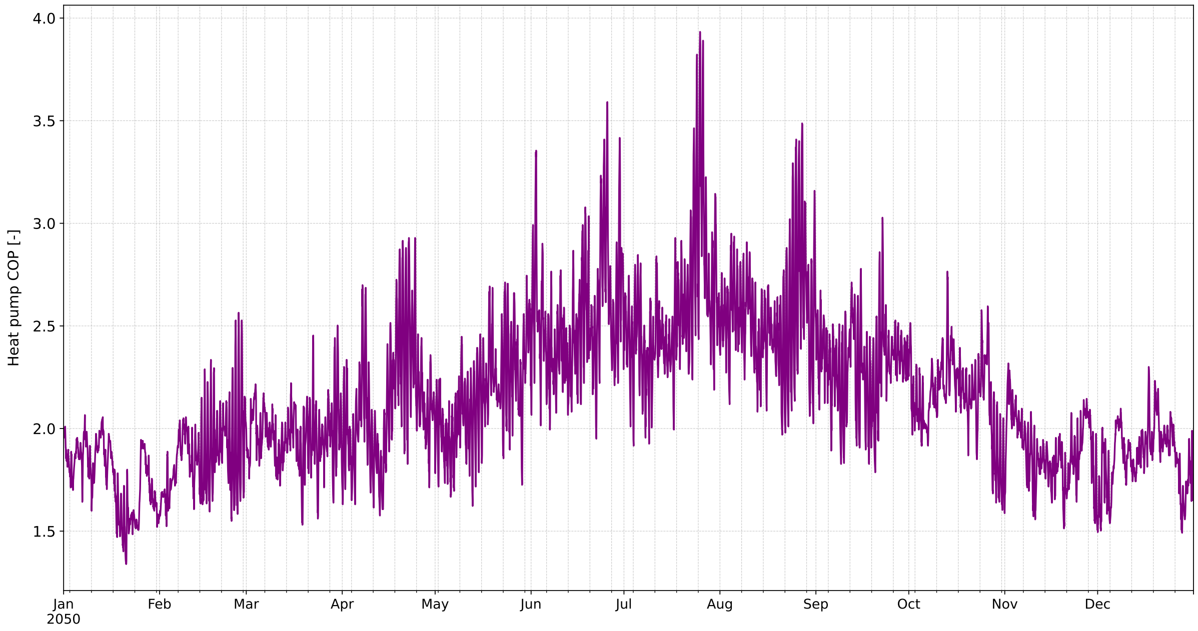

Figure S9. Air-source heat pump coefficient of performance

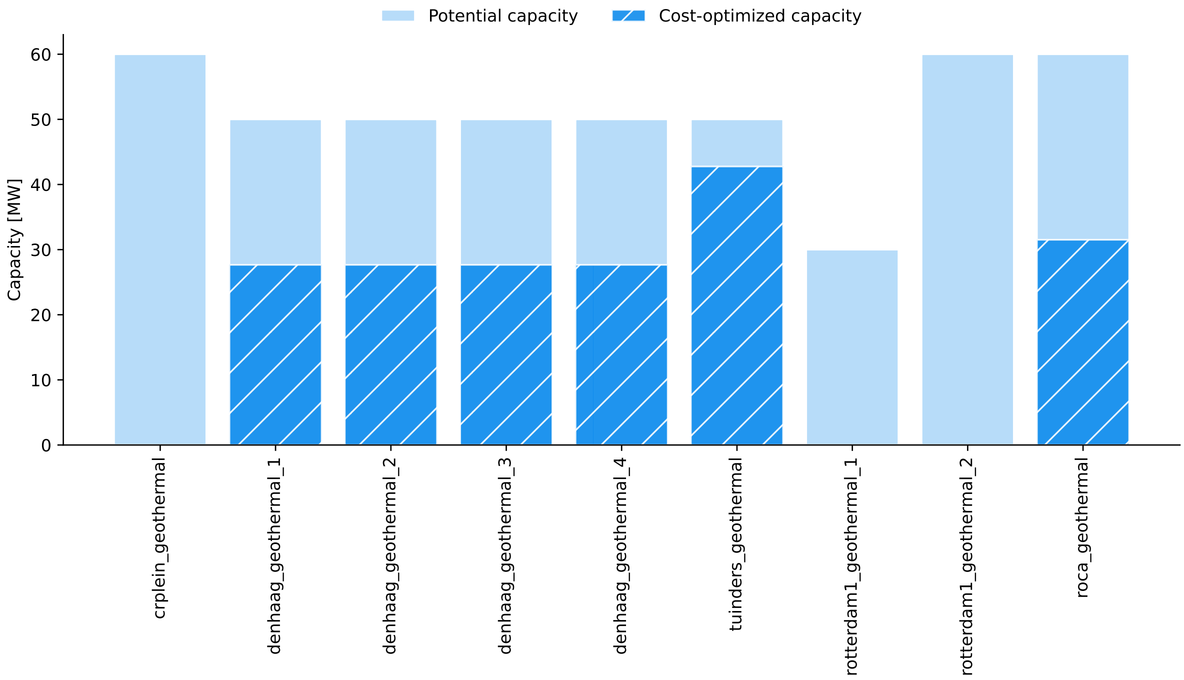

Figure S10. Spatial deployment of geothermal heat sources in the least-cost solution

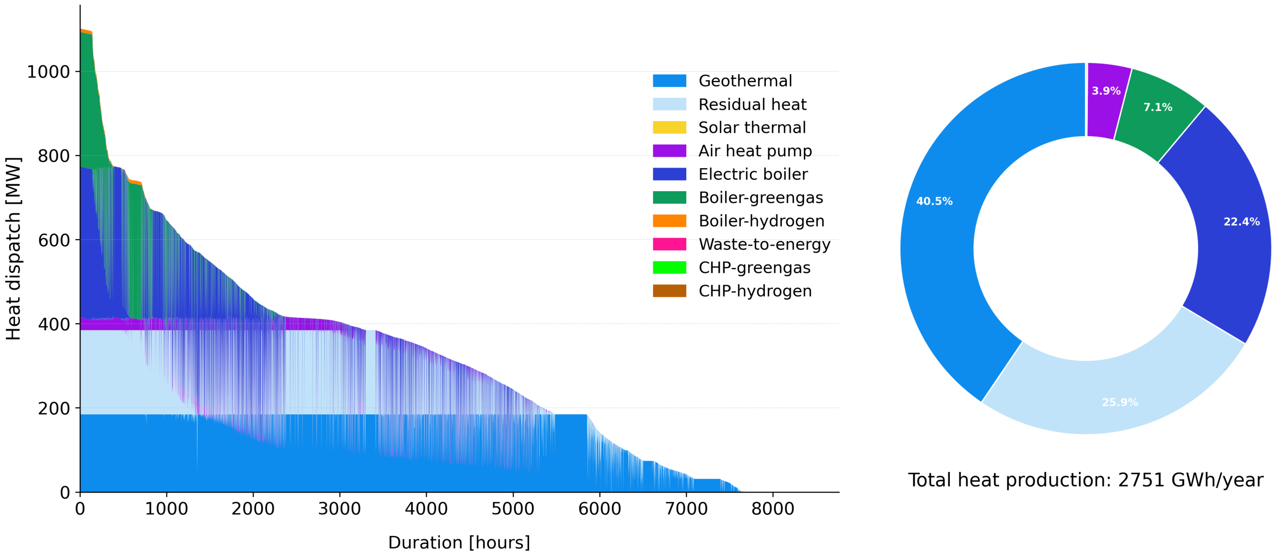

Figure S11. Heat dispatch duration curve and annual share of heat supply per technology category in the least-cost solution

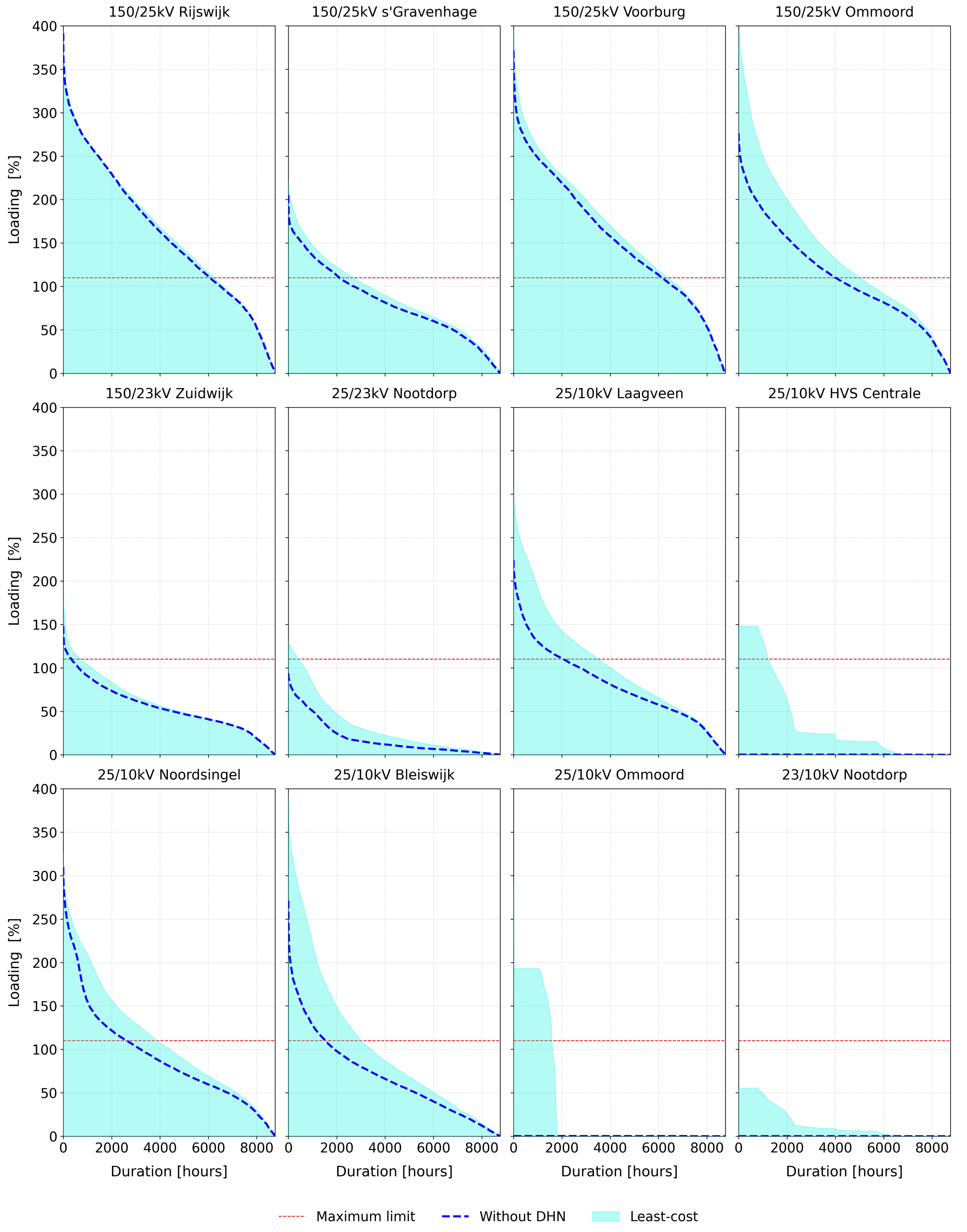

1374

Figure S12. Loading duration curves of all transformers in the electricity network

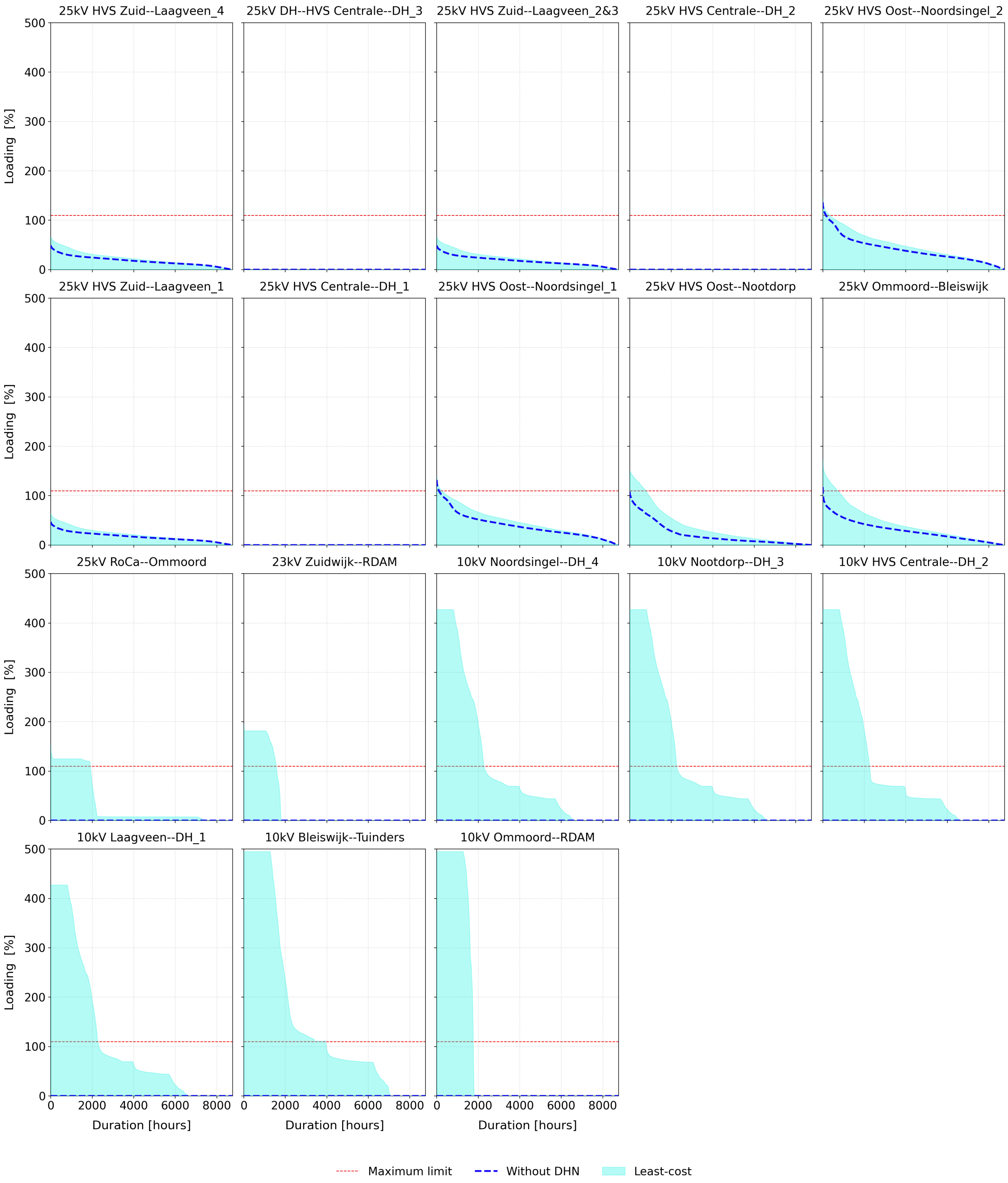

1375

Figure S13. Loading duration curves of all lines in the electricity network

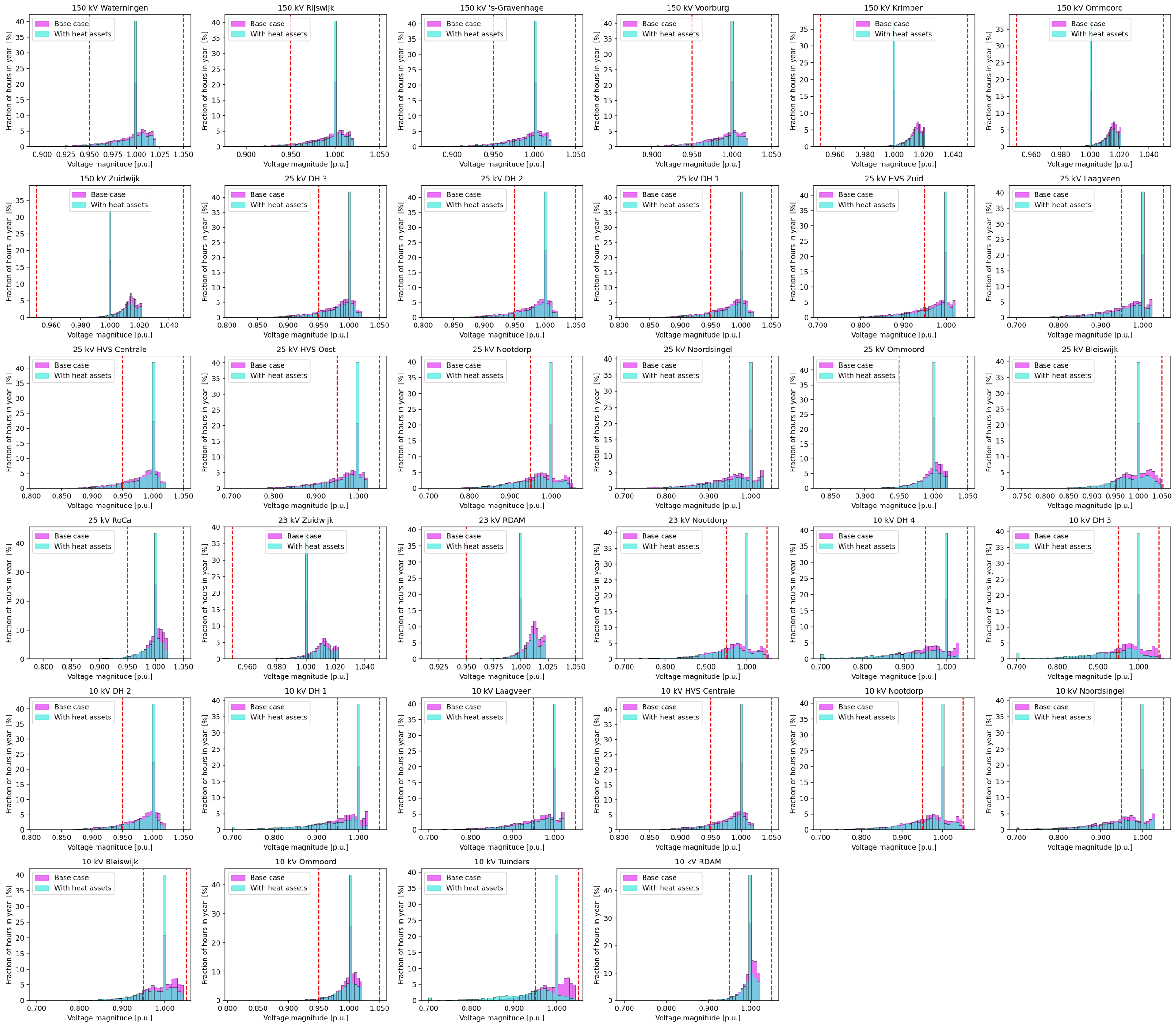

1376

Figure S14. Voltage magnitude histograms of all buses in the electricity network

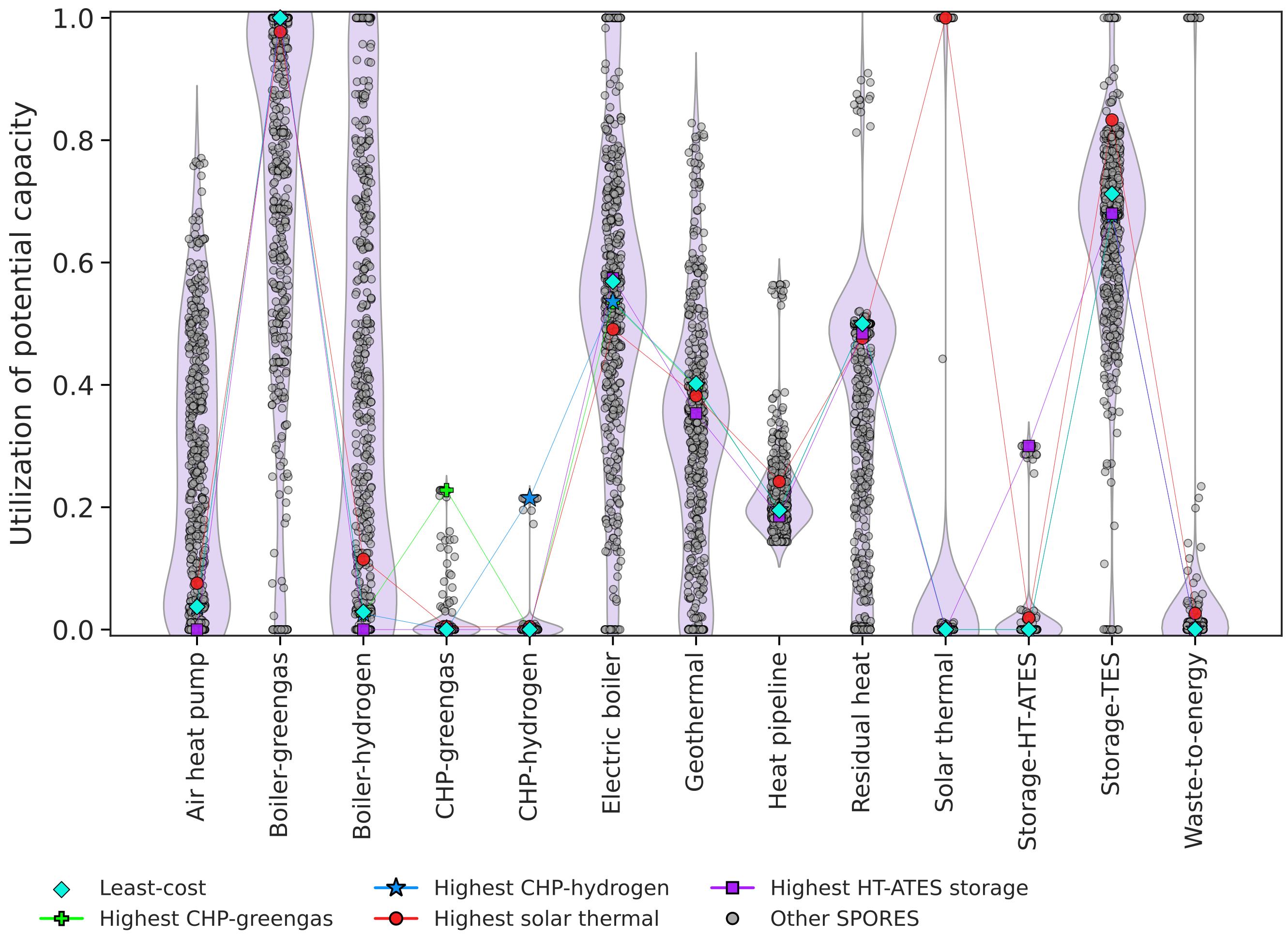

1377

Figure S15. Trade-offs associated with maximizing technologies with low-utilization frequencies across SPORES

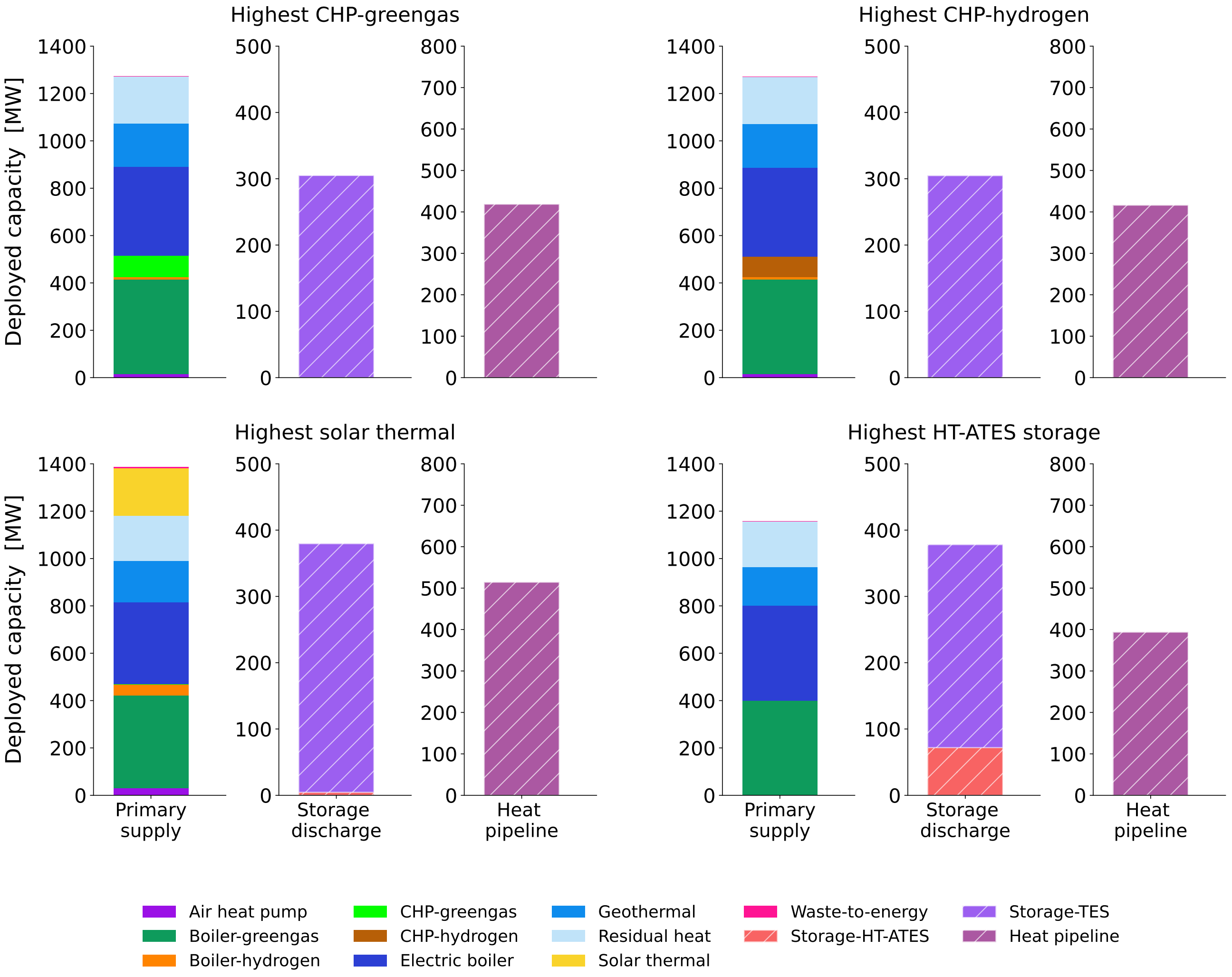

1378

Figure S16. Technology configurations under maximum build-out of technologies with low-utilization frequencies across SPORES

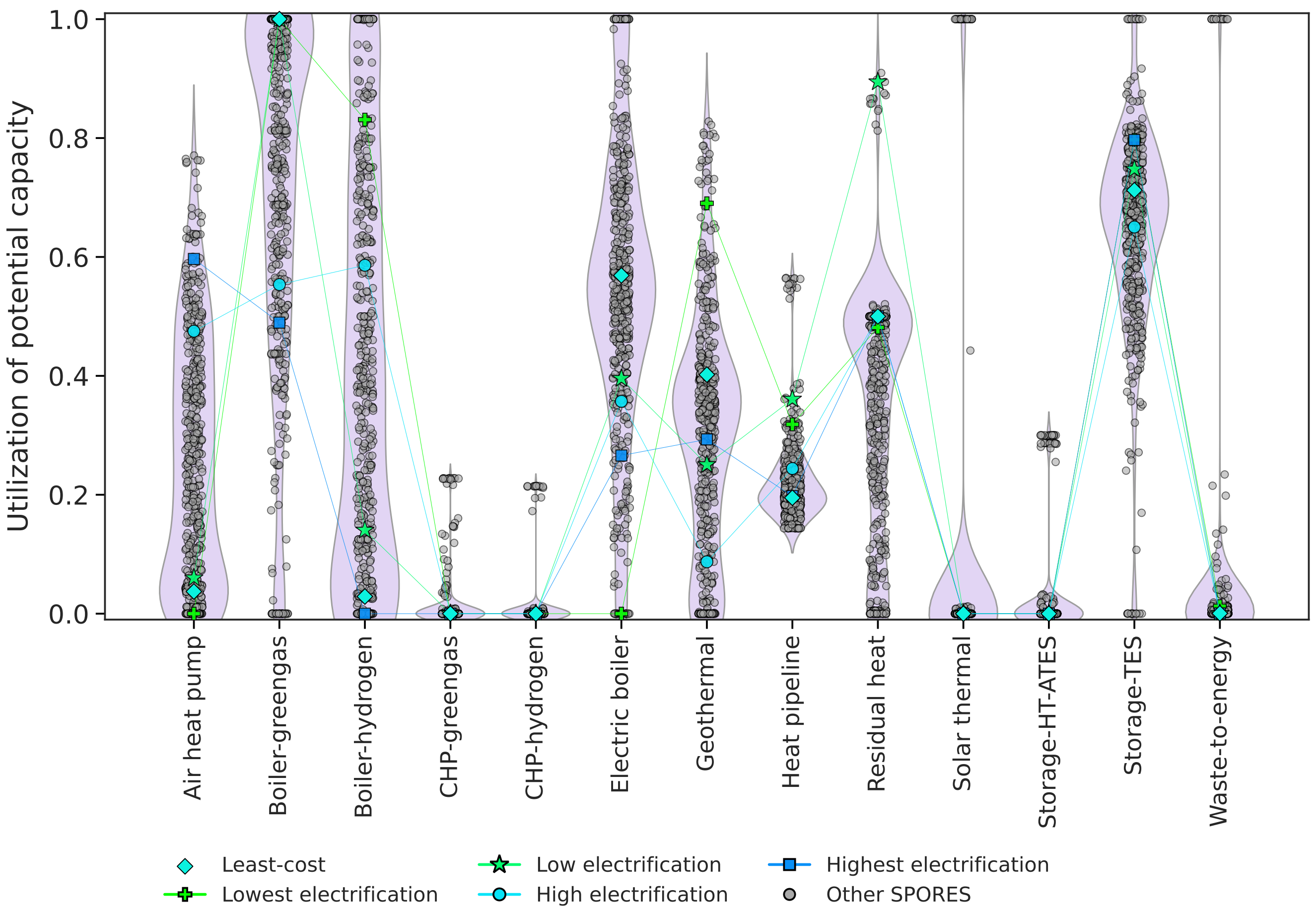

Figure S17. Trade-offs across highlighted SPORES with varying degrees of heat electrification

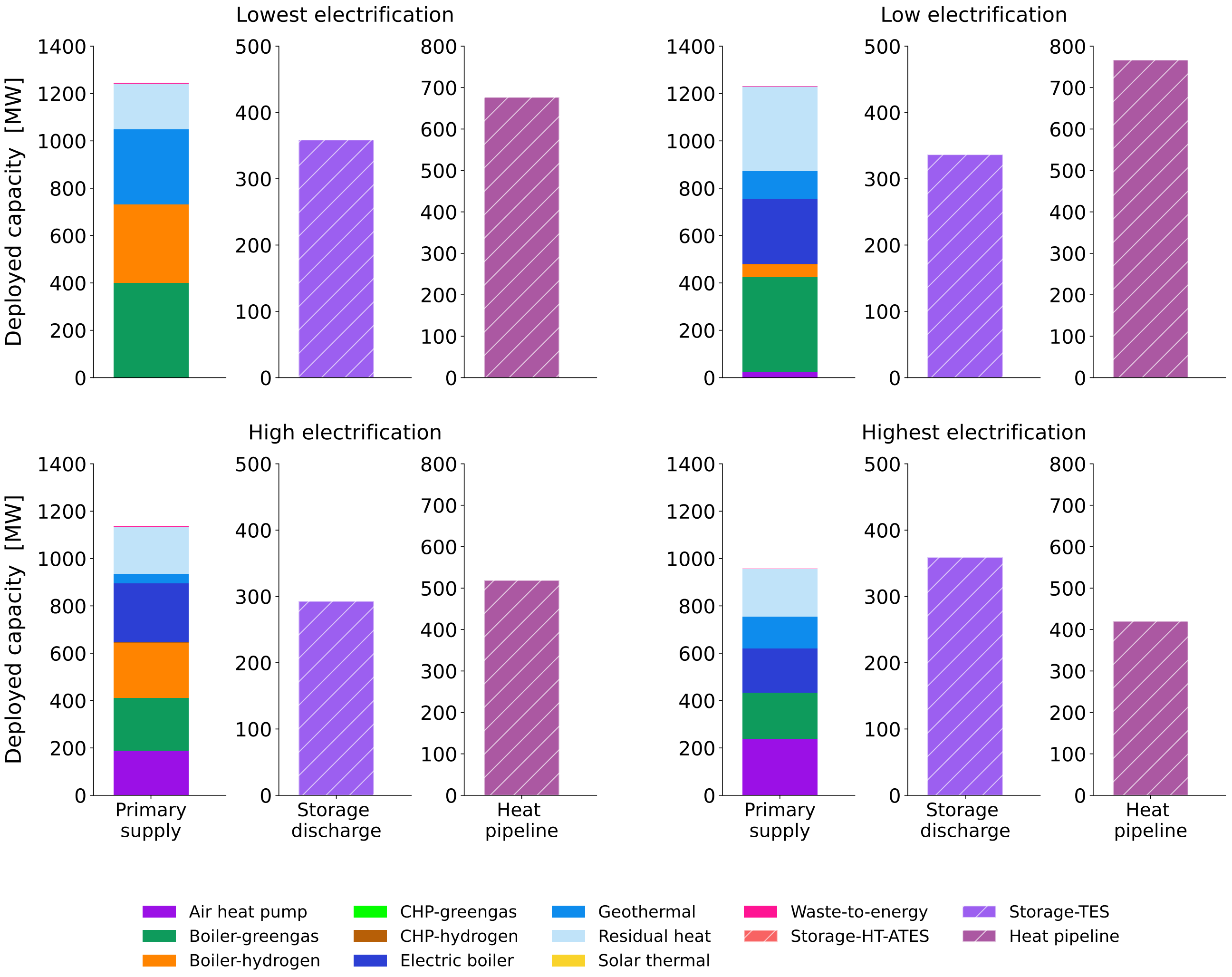

Figure S18. Technology configurations for highlighted SPORES with varying degrees of heat electrification

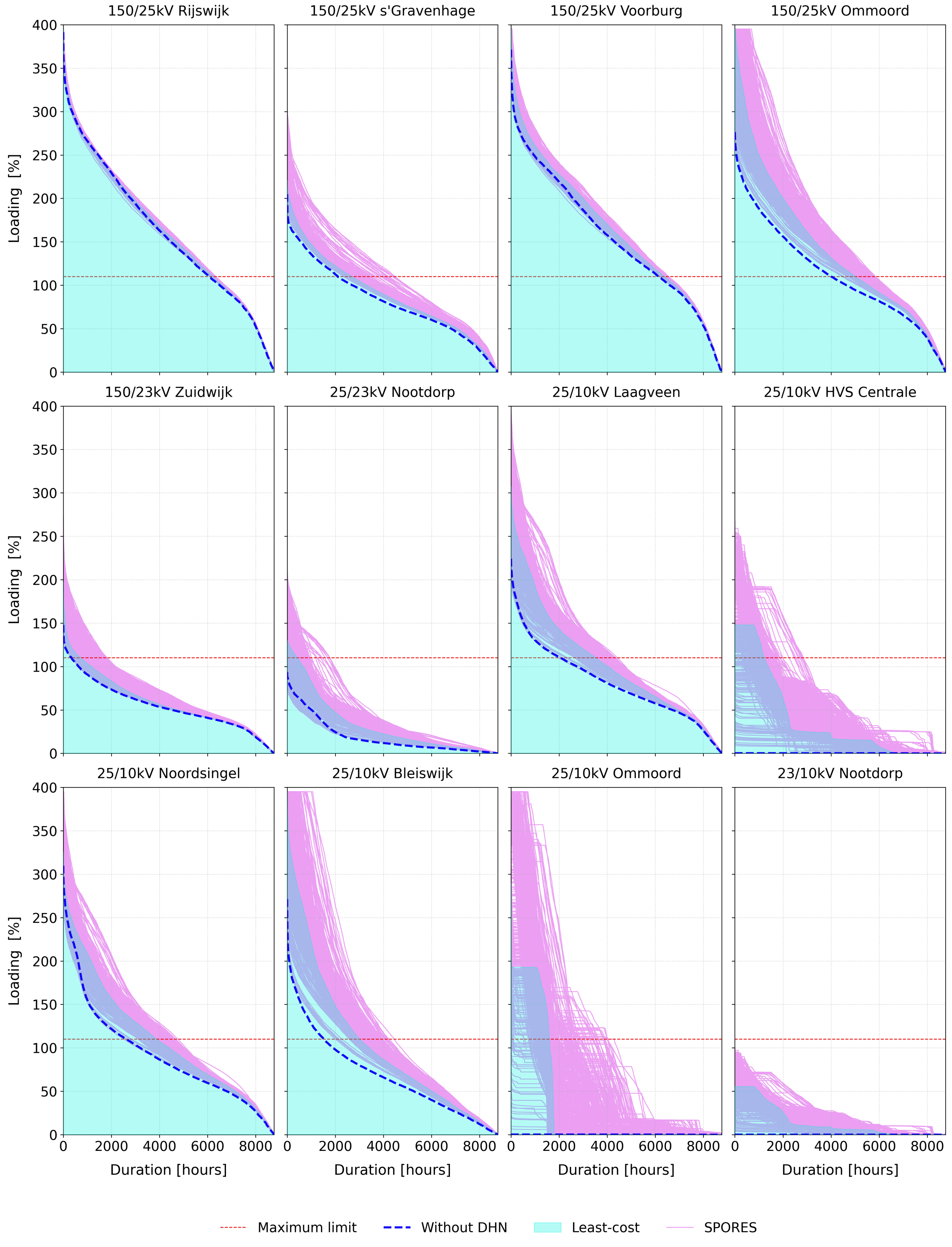

1381

Figure S19. Range of transformer loading duration across all SPORES

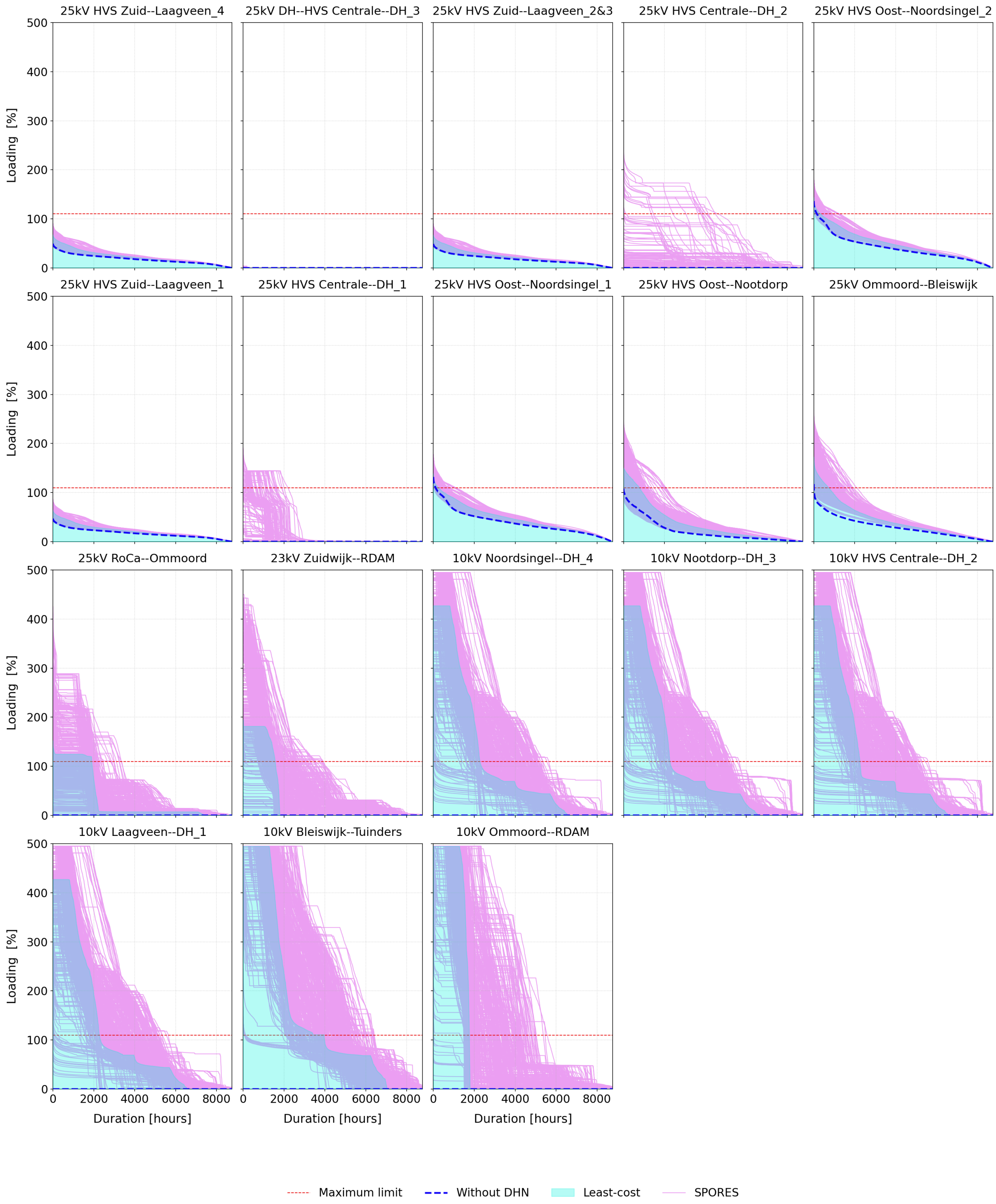

1382

Figure S20. Range of line loading duration across all SPORES

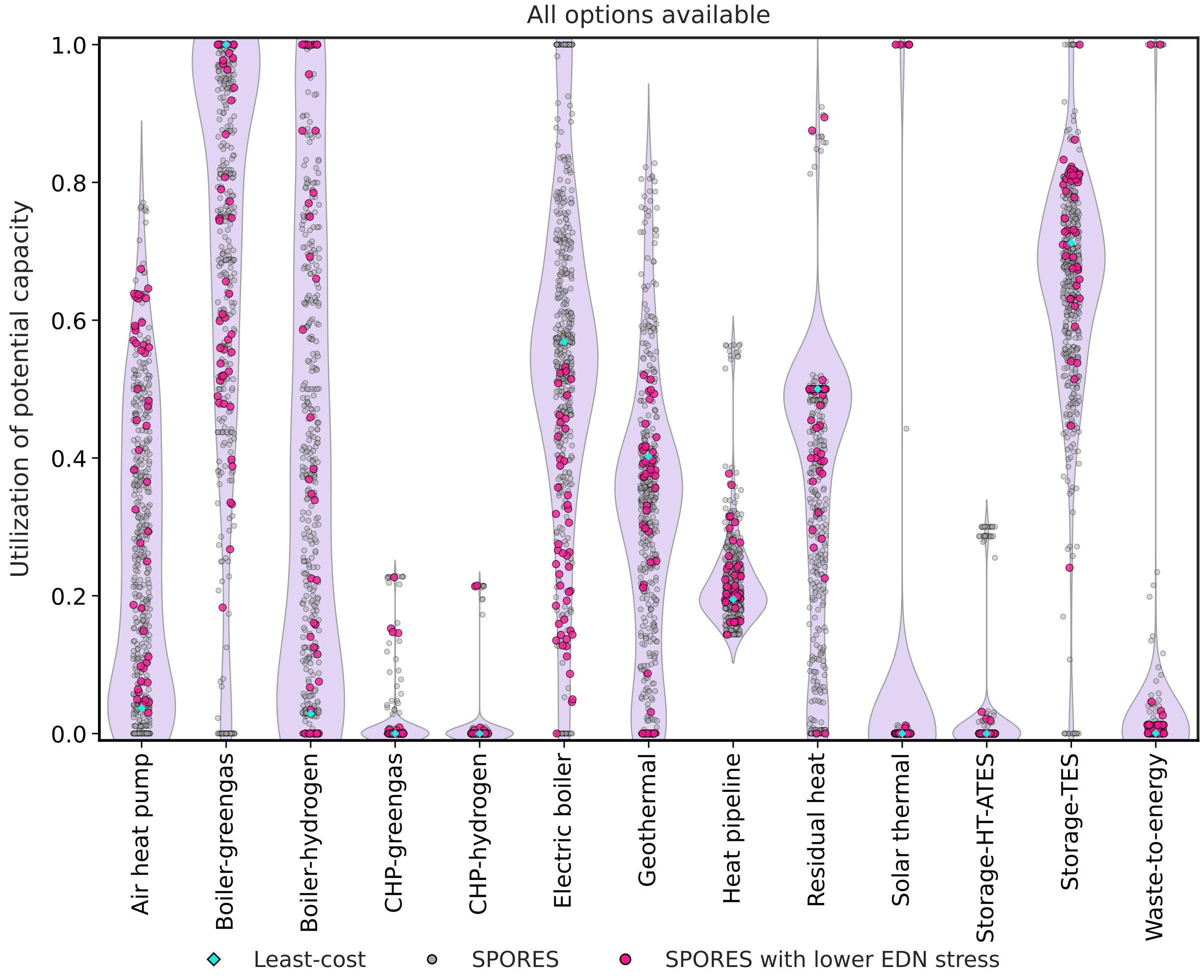

Figure S21. Frequency distribution of potential capacity utilization in the near-optimal decision space with lower grid loading SPORES highlighted: default scenario with 10% cost slack

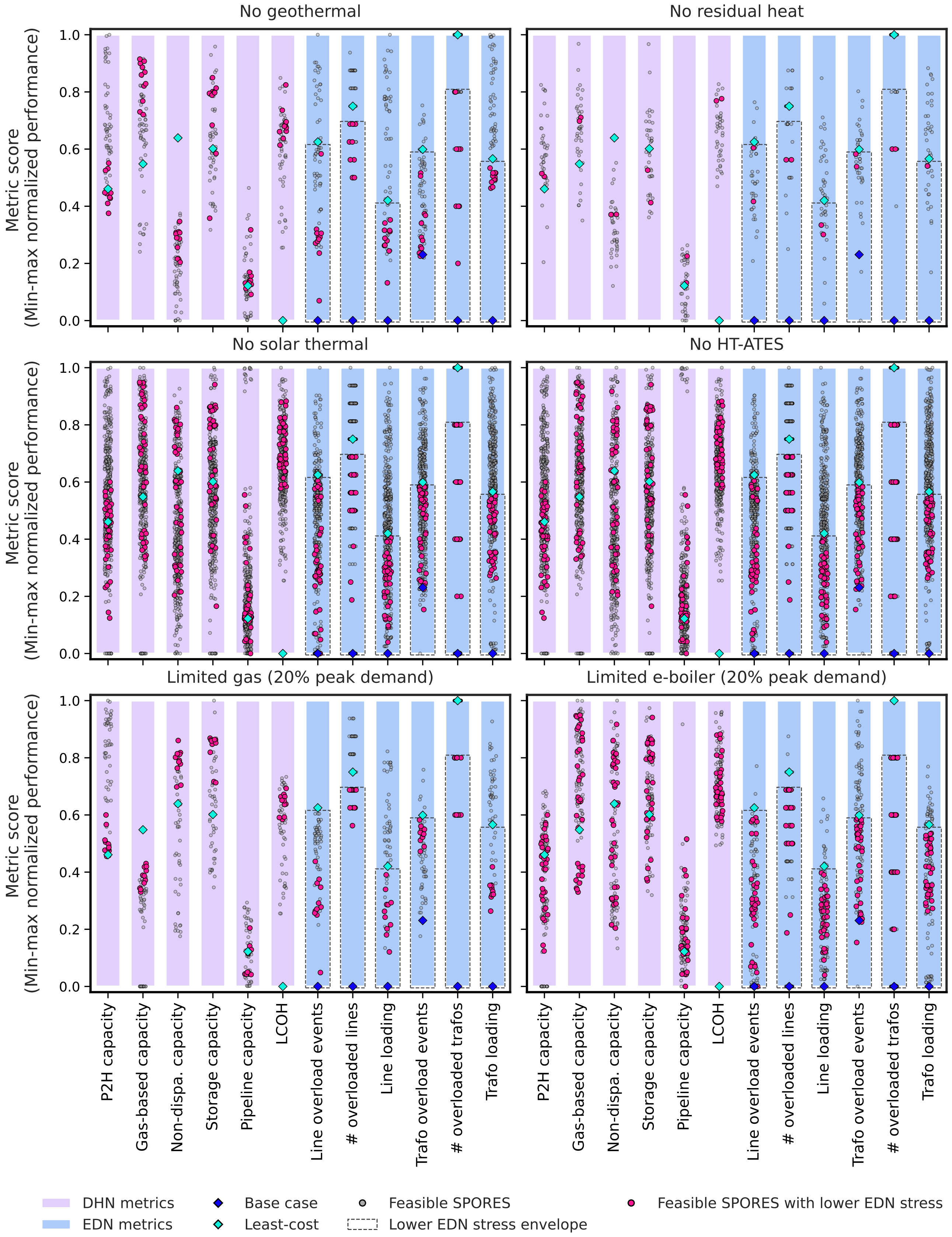

1384

Figure S22. Integrated decision space under technology deployment constraints: default scenario with 10% cost slack

Sensitivity analysis results

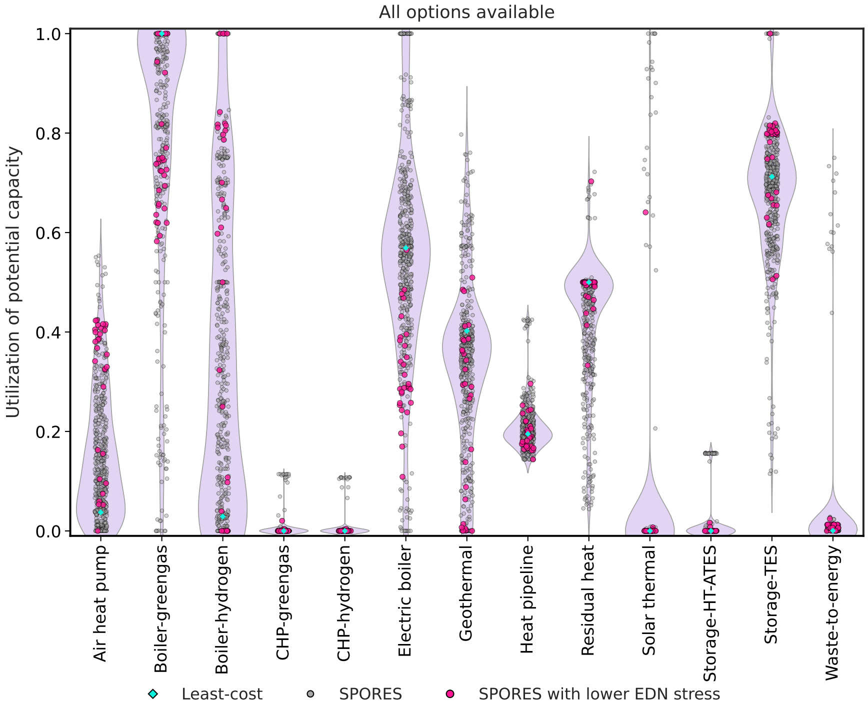

Figure S23. Frequency distribution of potential capacity utilization in the near-optimal decision space with lower grid loading SPORES highlighted: 5% cost slack scenario

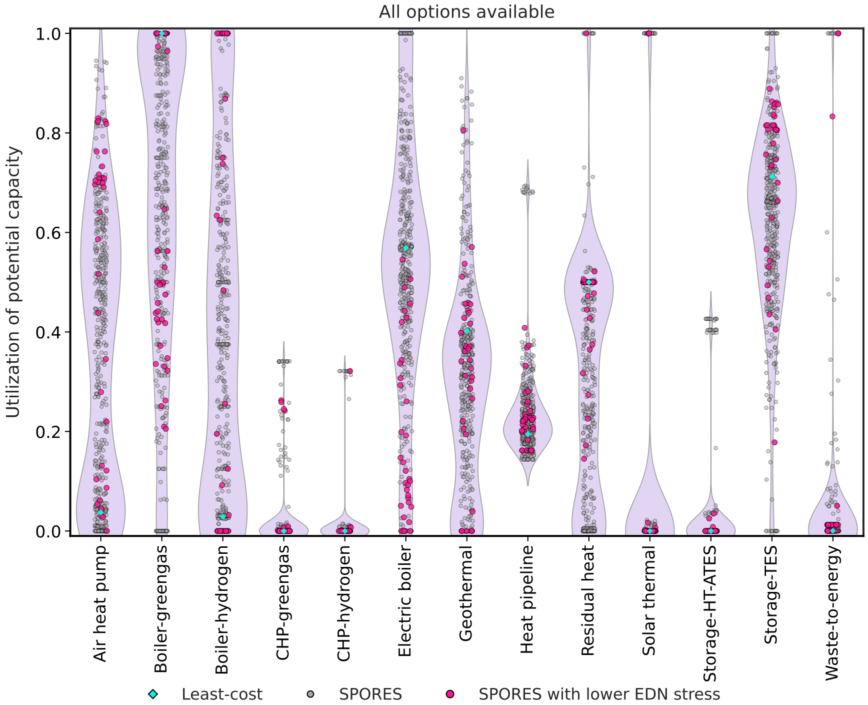

Figure S24. Frequency distribution of potential capacity utilization in the near-optimal decision space with lower grid loading SPORES highlighted: 15% cost slack scenario

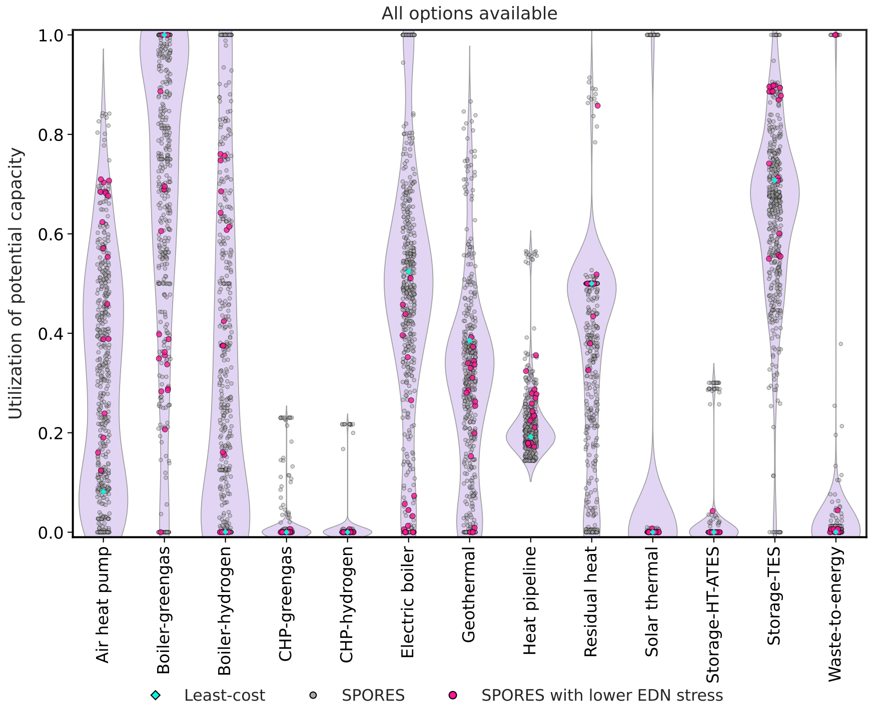

Figure S25. Frequency distribution of potential capacity utilization in the near-optimal decision space with lower grid loading SPORES highlighted: warm weather year scenario

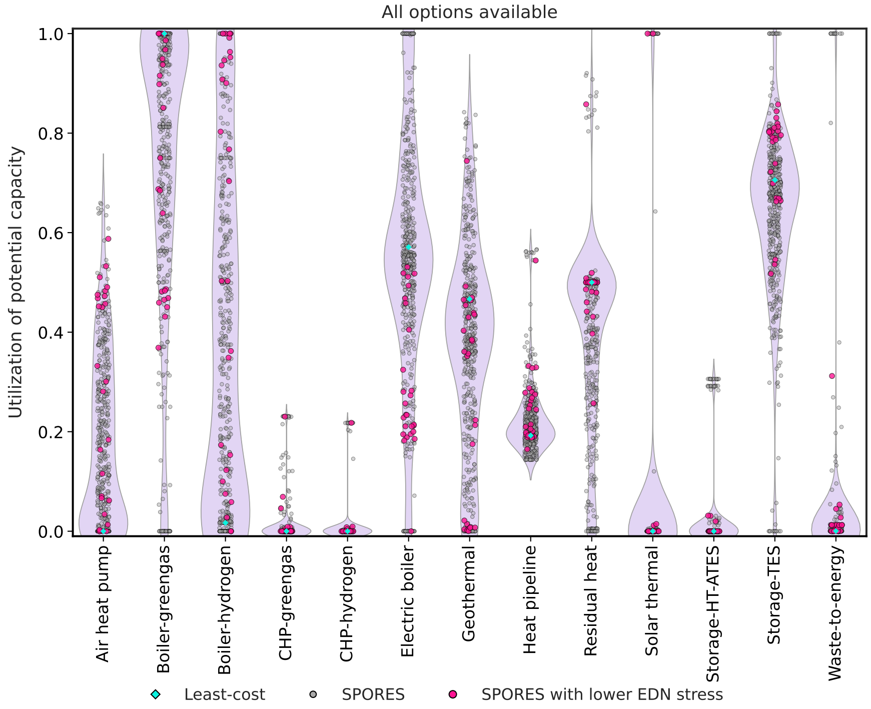

Figure S26. Frequency distribution of potential capacity utilization in the near-optimal decision space with lower grid loading SPORES highlighted: cold weather year scenario

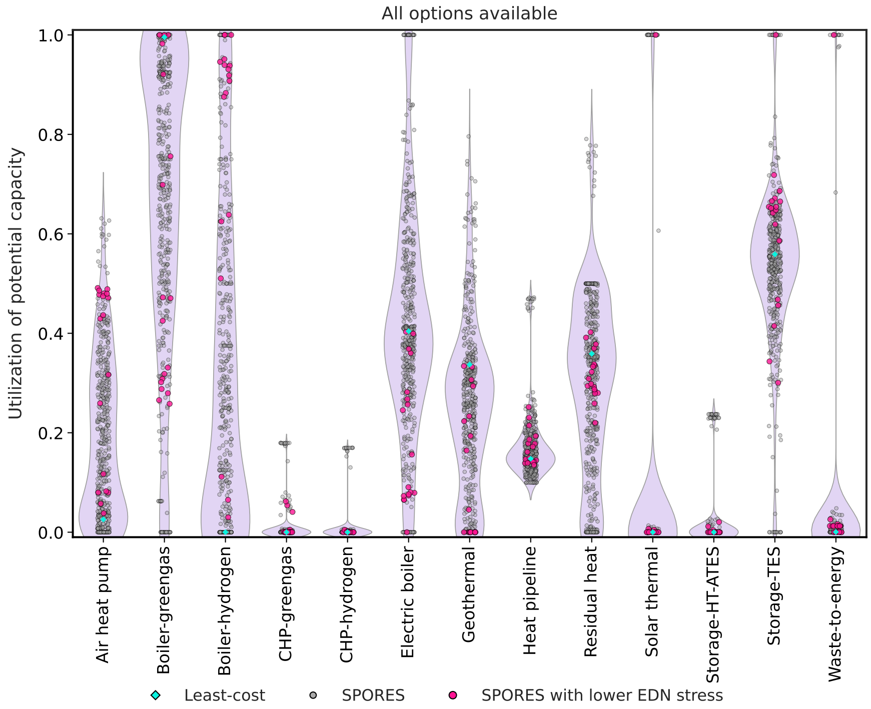

Figure S27. Frequency distribution of potential capacity utilization in the near-optimal decision space with lower grid loading SPORES highlighted: low heat demand scenario

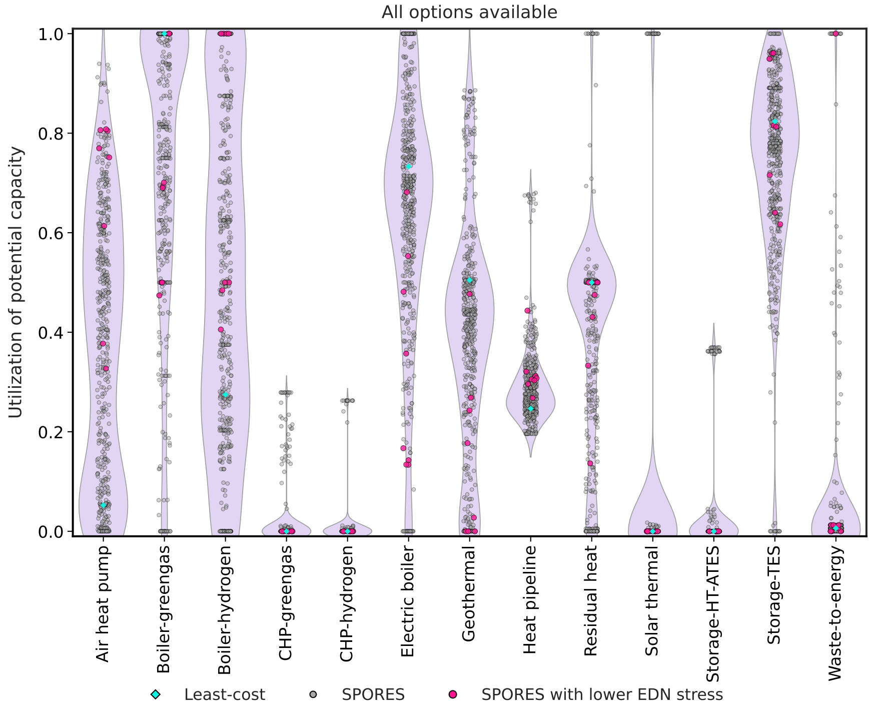

Figure S28. Frequency distribution of potential capacity utilization in the near-optimal decision space with lower grid loading SPORES highlighted: high heat demand scenario

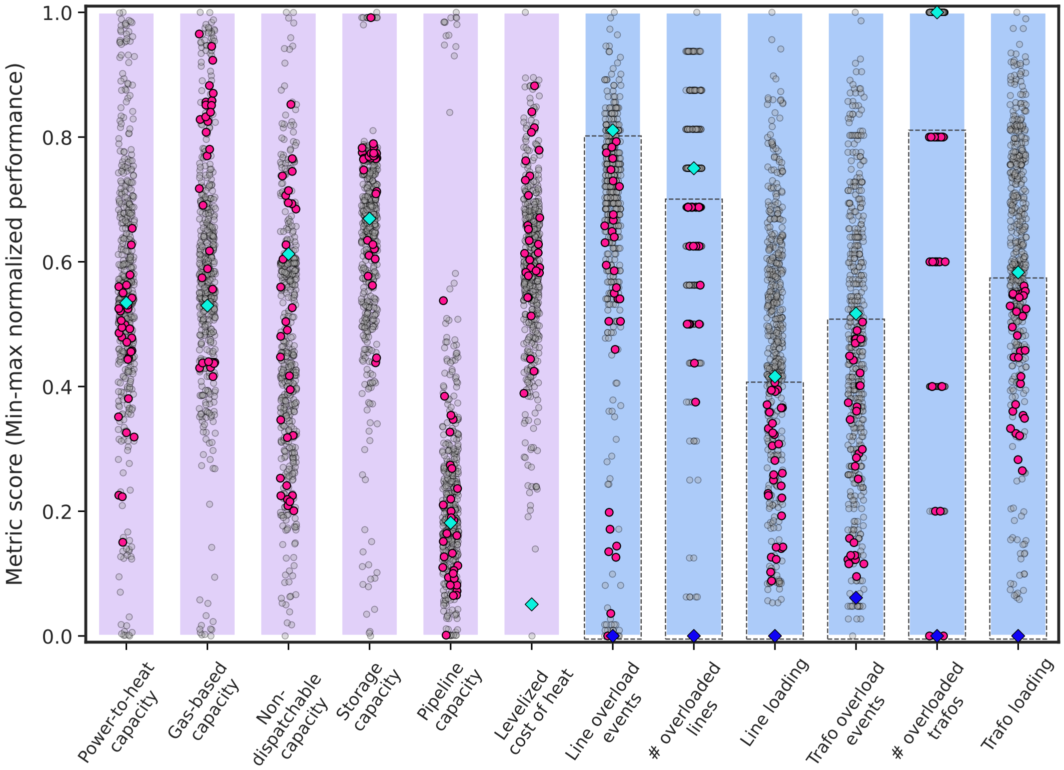

Figure S29. Integrated decision space: 5% cost slack scenario

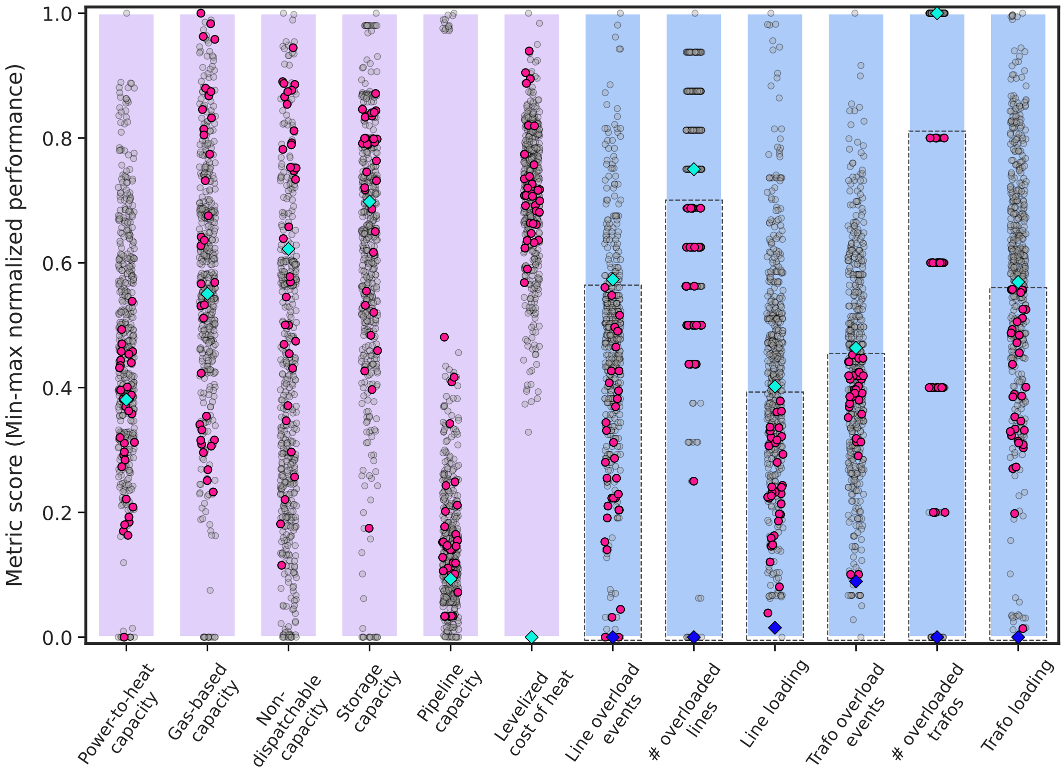

Figure S30. Integrated decision space: 15% cost slack scenario

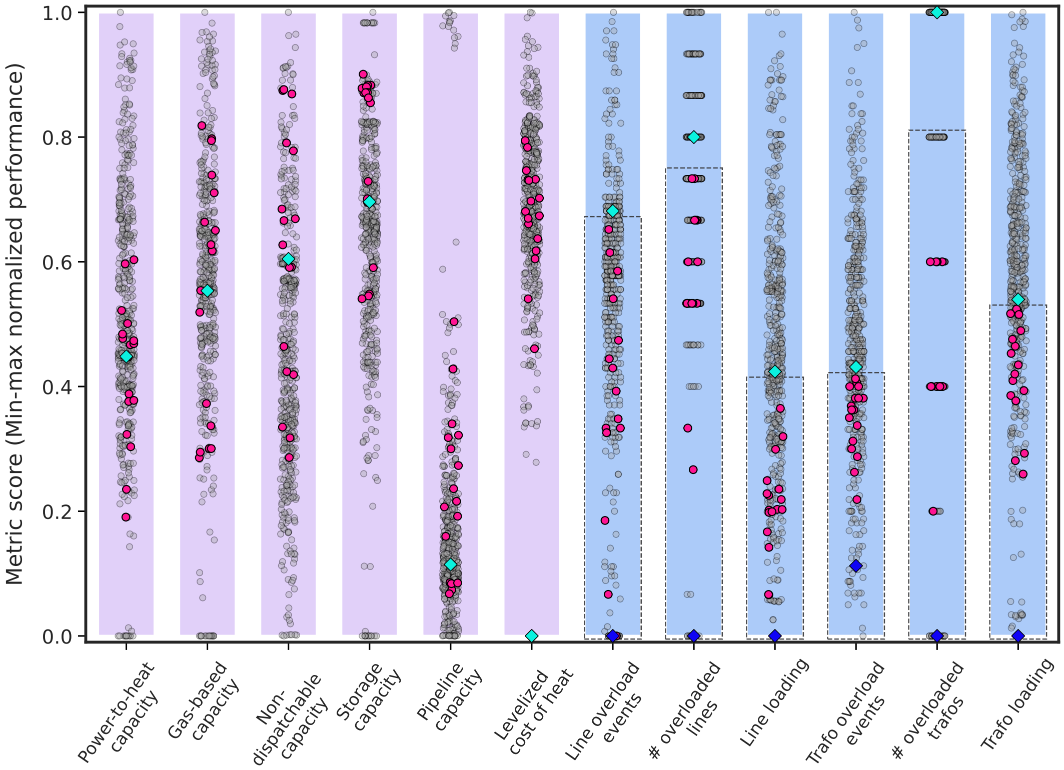

Figure S31. Integrated decision space: warm weather year scenario

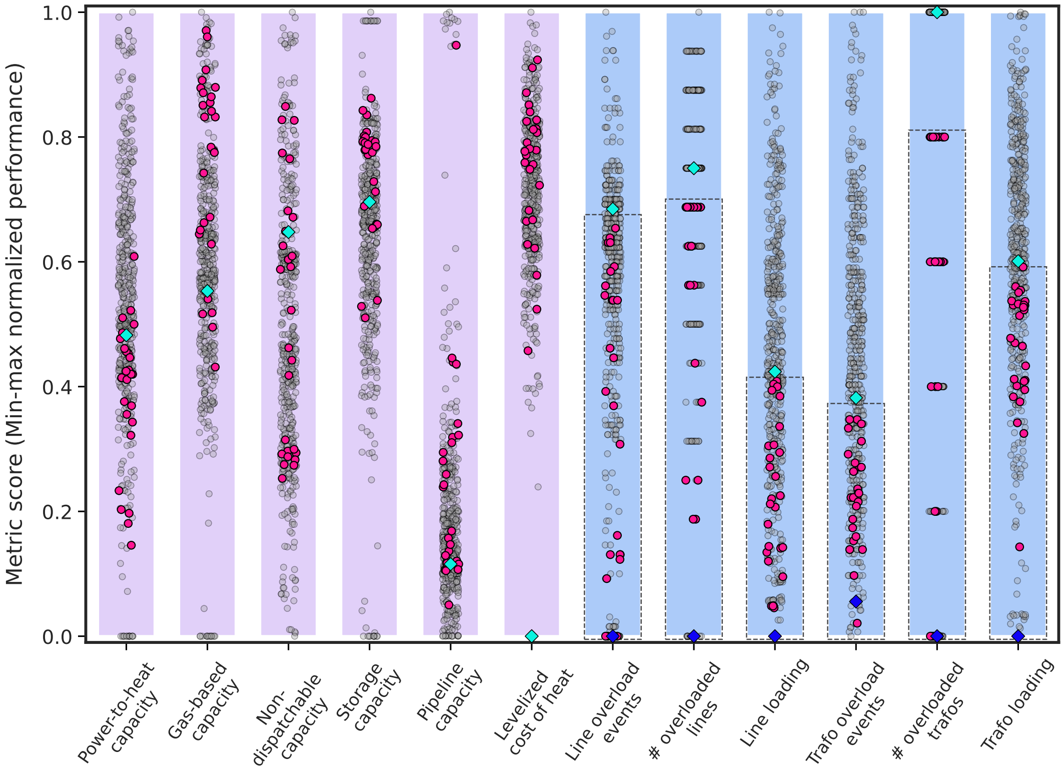

Figure S32. Integrated decision space: cold weather year scenario

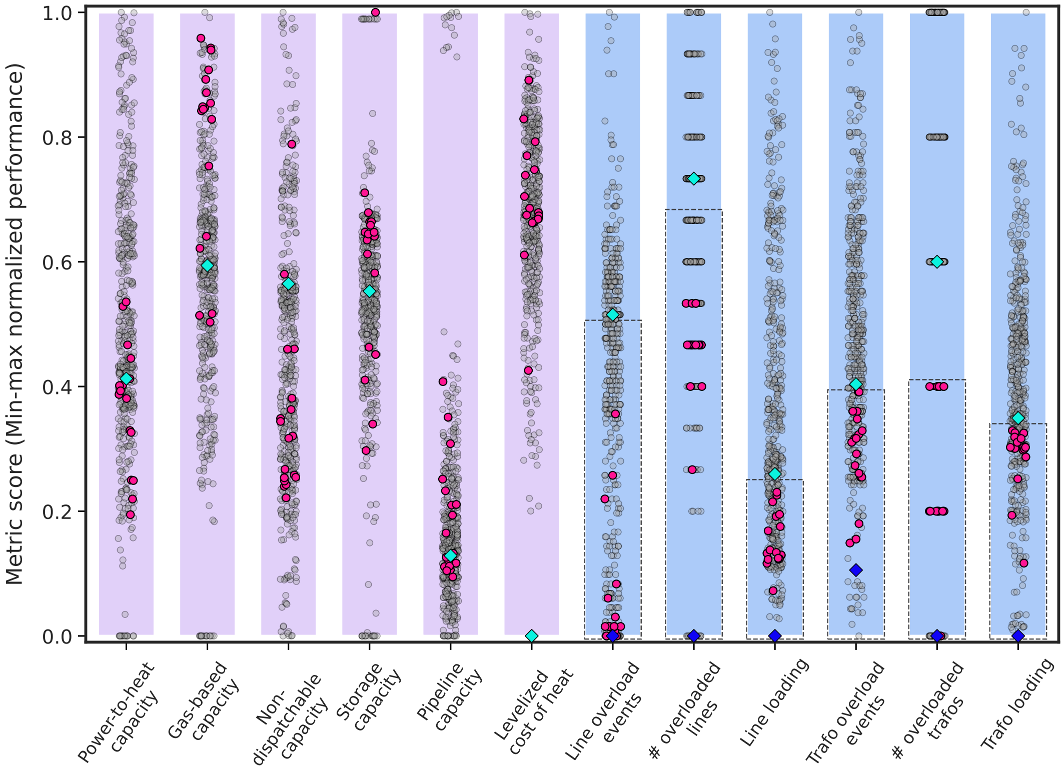

Figure S33. Integrated decision space: low heat demand scenario

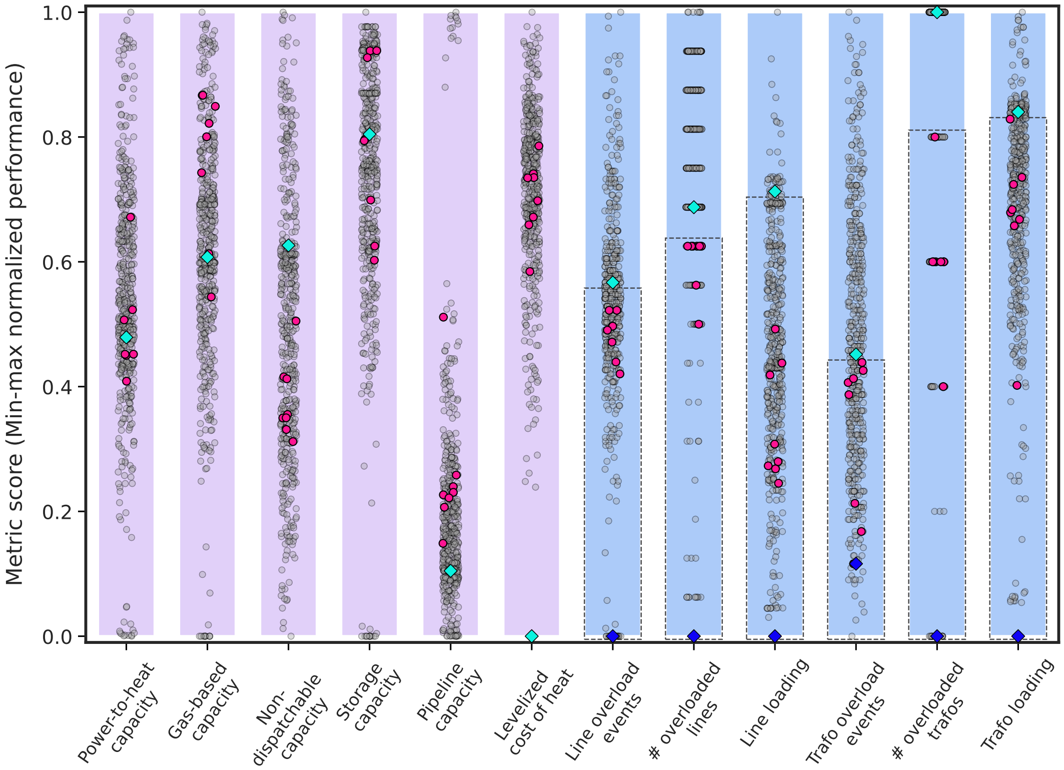

Figure S34. Integrated decision space: high heat demand scenario

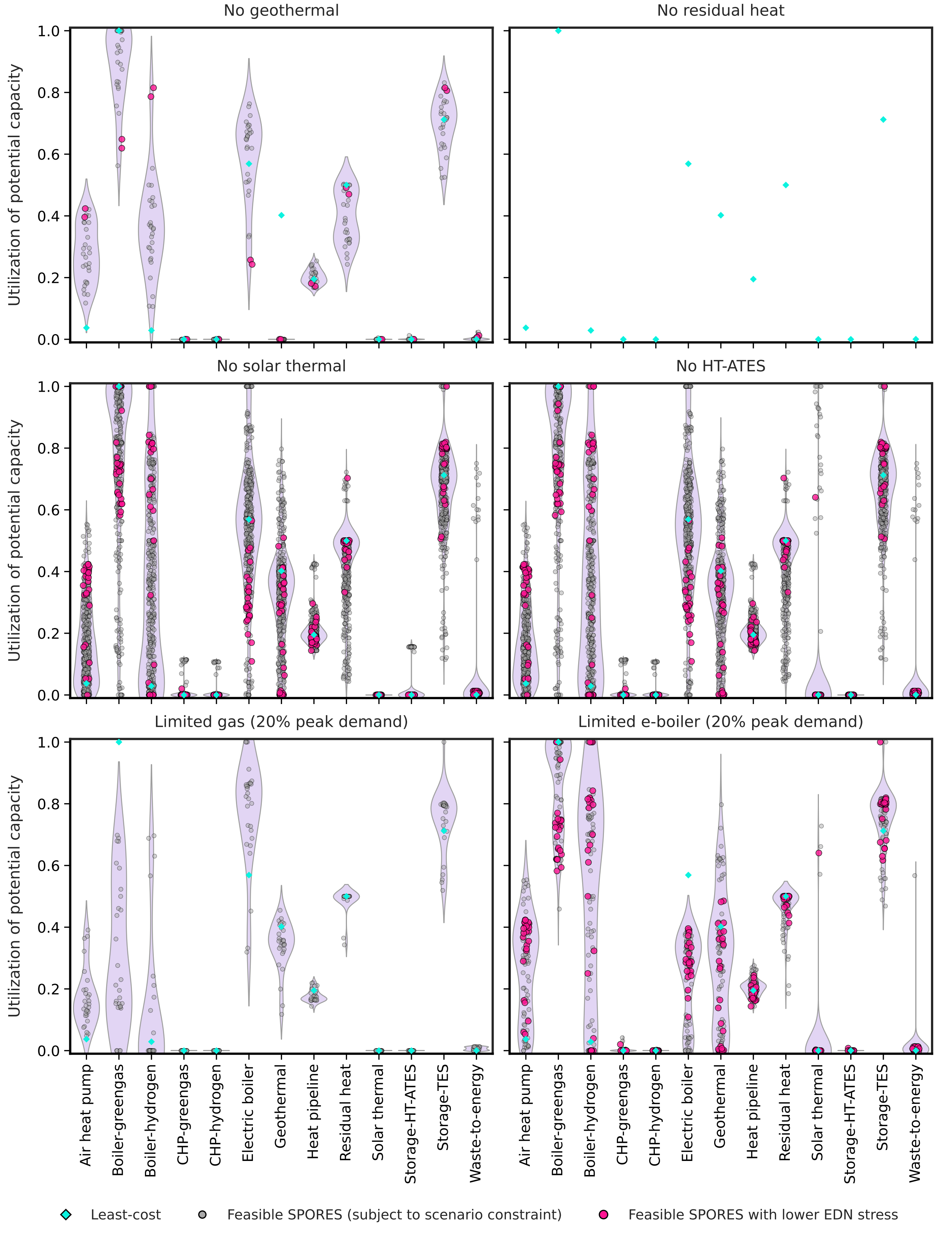

1398

Figure S35. Trade-offs under local technology deployment constraints: 5% cost slack scenario

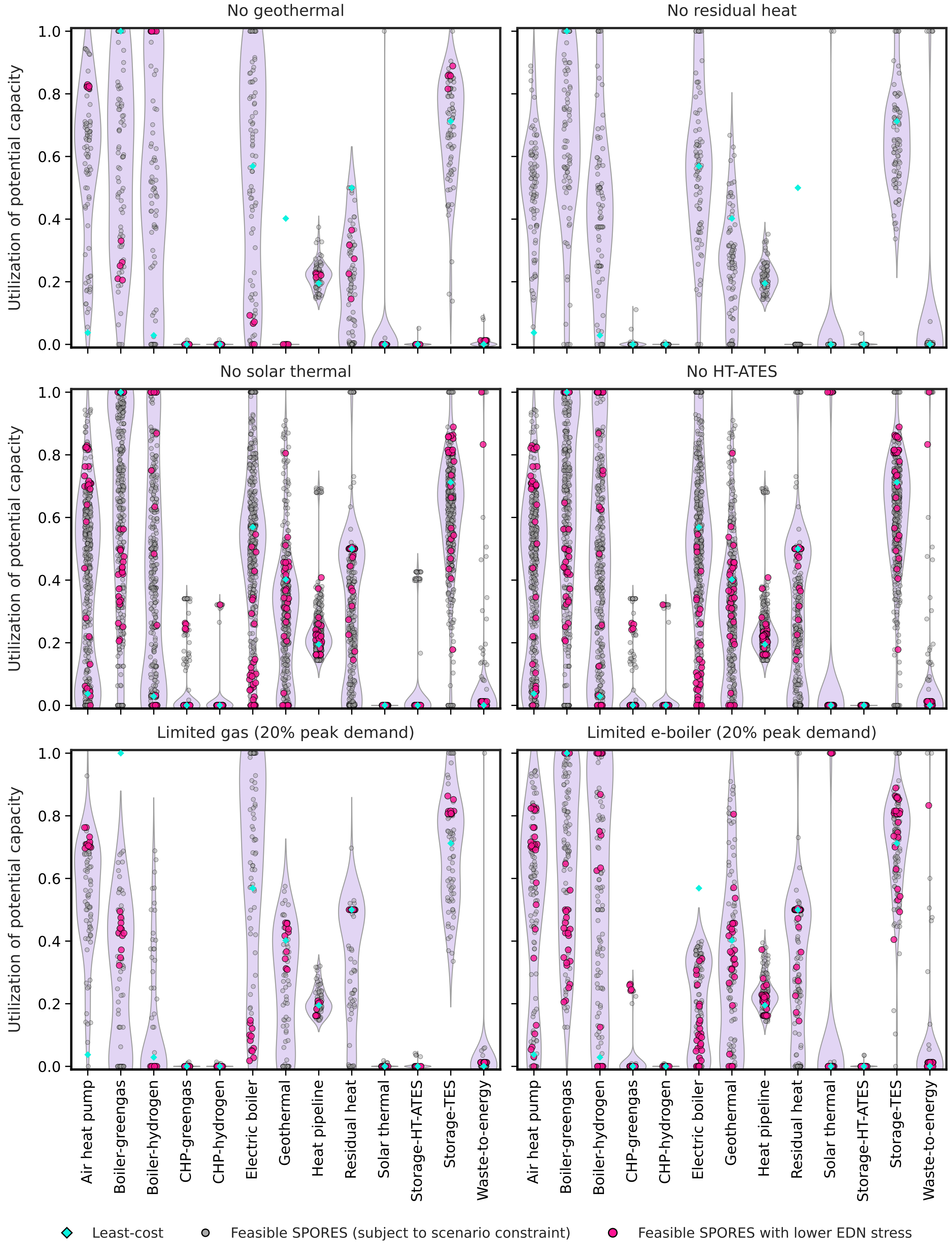

1399

Figure S36. Trade-offs under local technology deployment constraints: 15% cost slack scenario

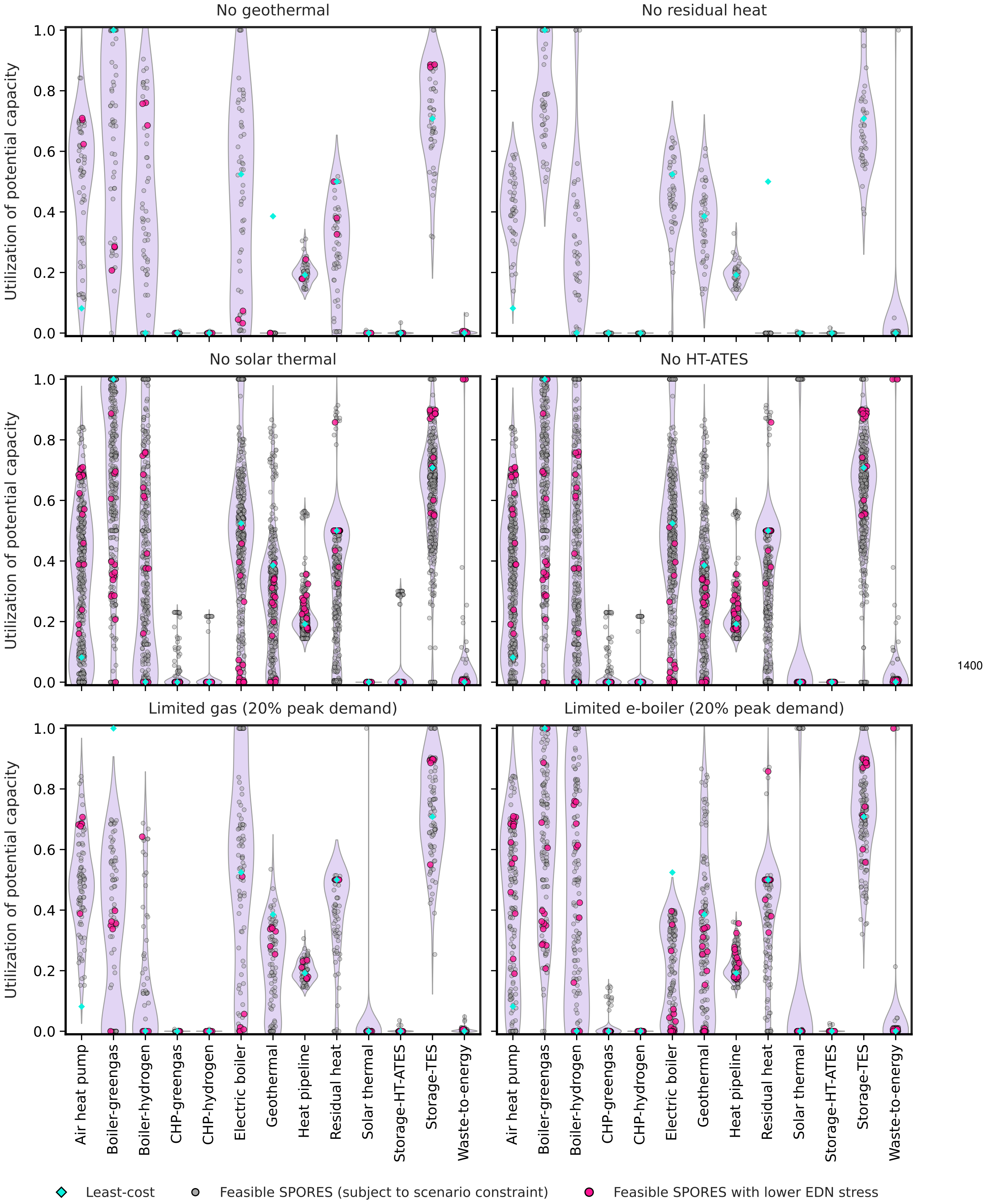

Figure S37. Trade-offs under local technology deployment constraints: warm weather year scenario

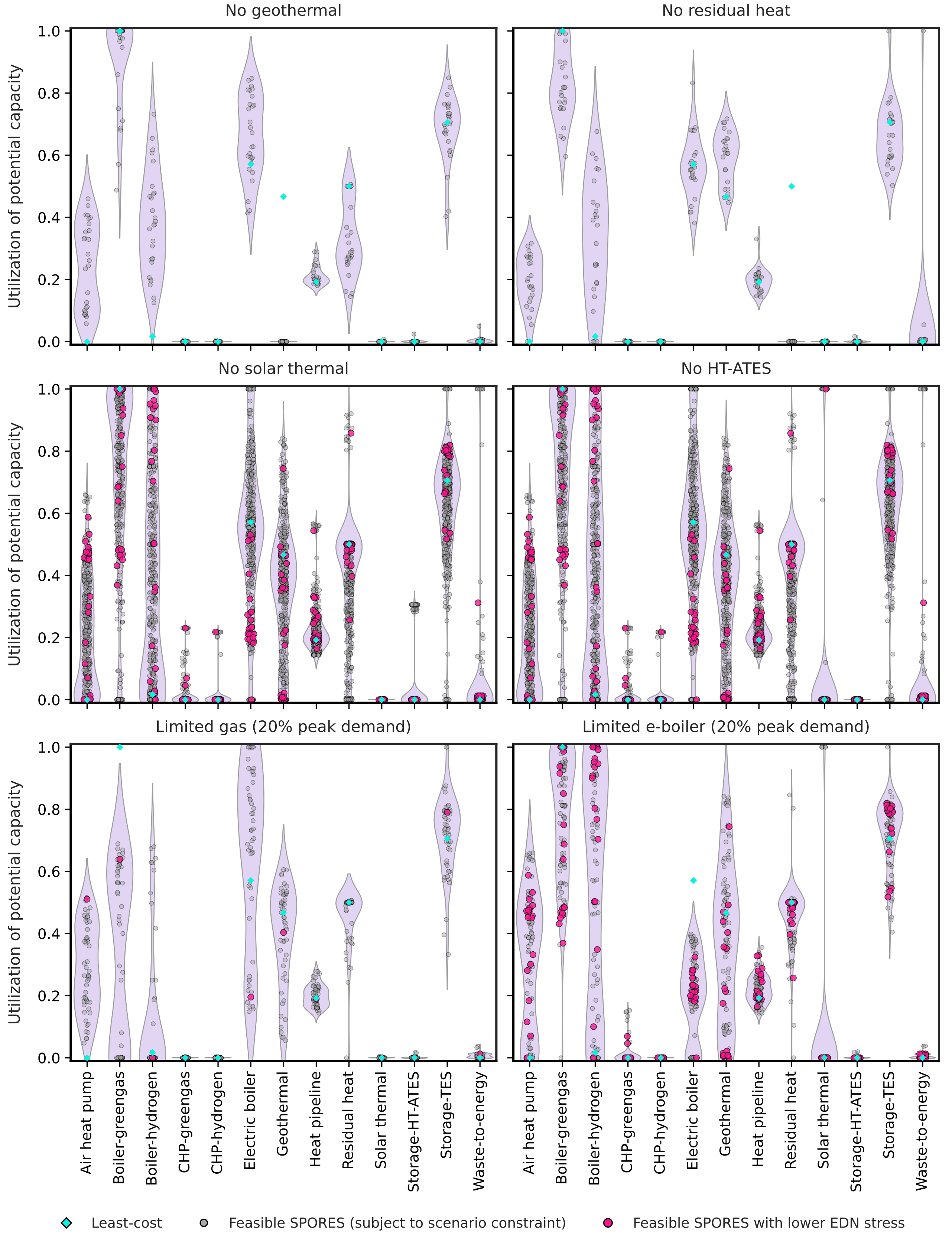

1401

Figure S38. Trade-offs under local technology deployment constraints: cold weather year scenario

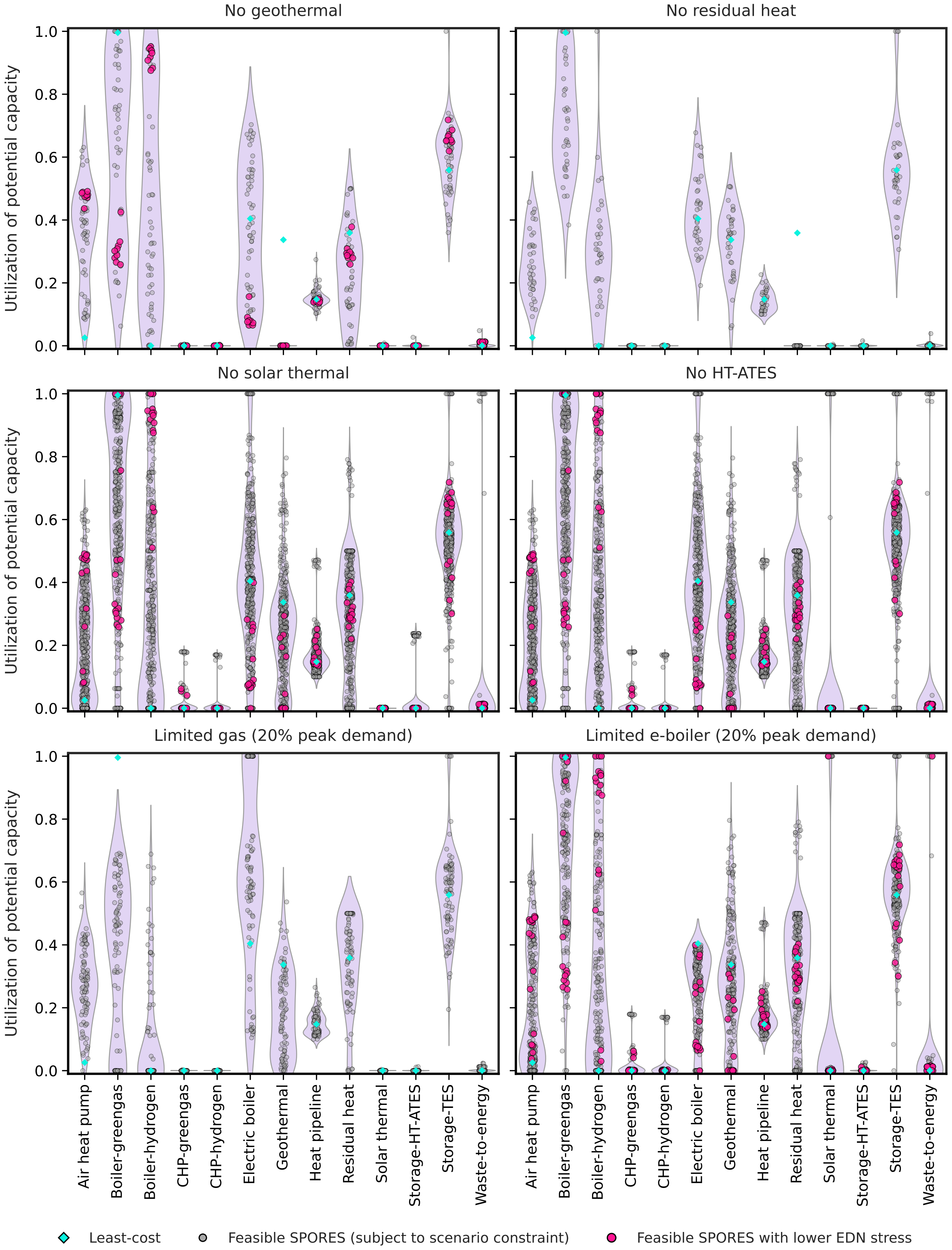

1402

Figure S39. Trade-offs under local technology deployment constraints: low heat demand scenario

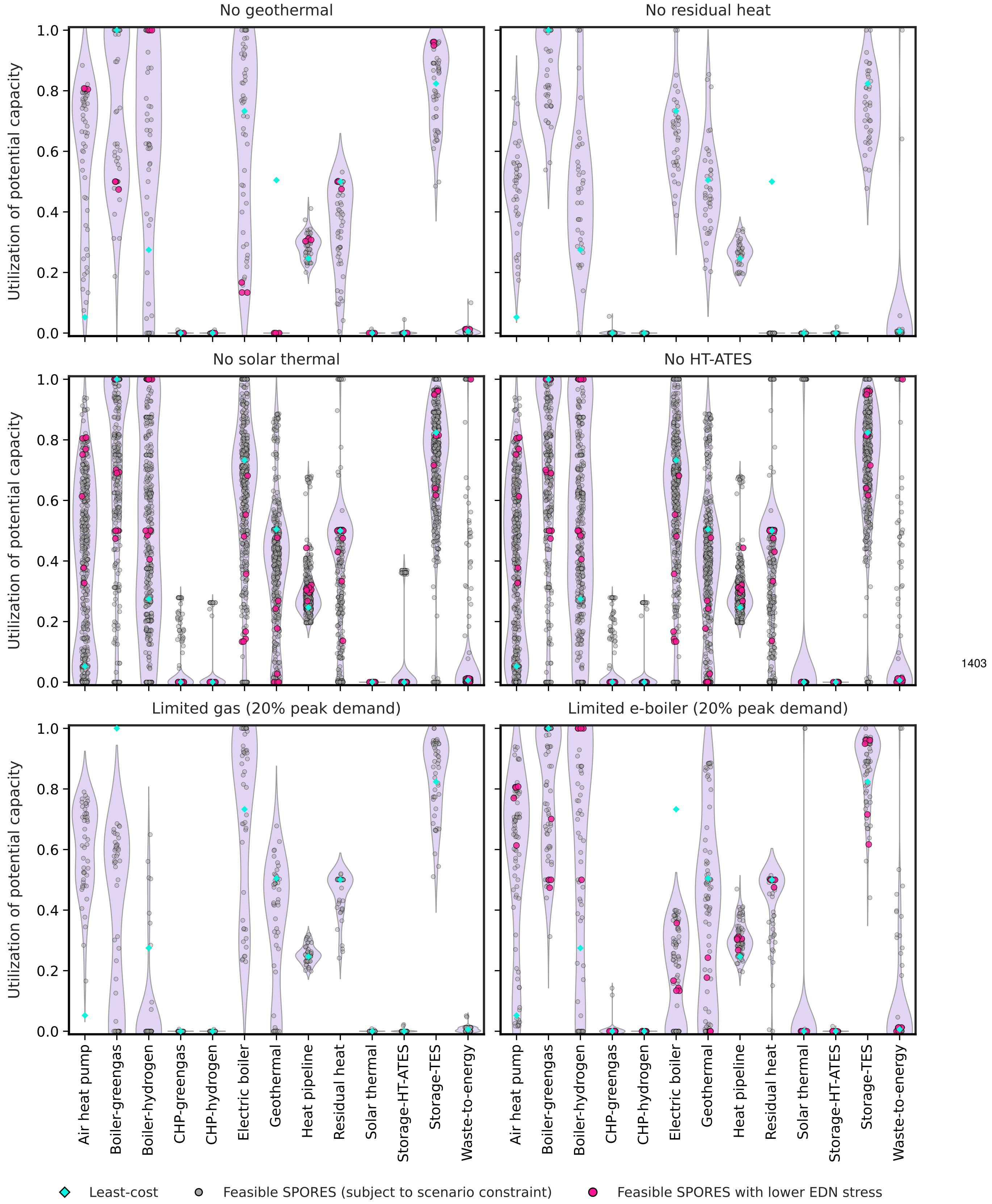

Figure S40. Trade-offs under local technology deployment constraints: high heat demand scenario

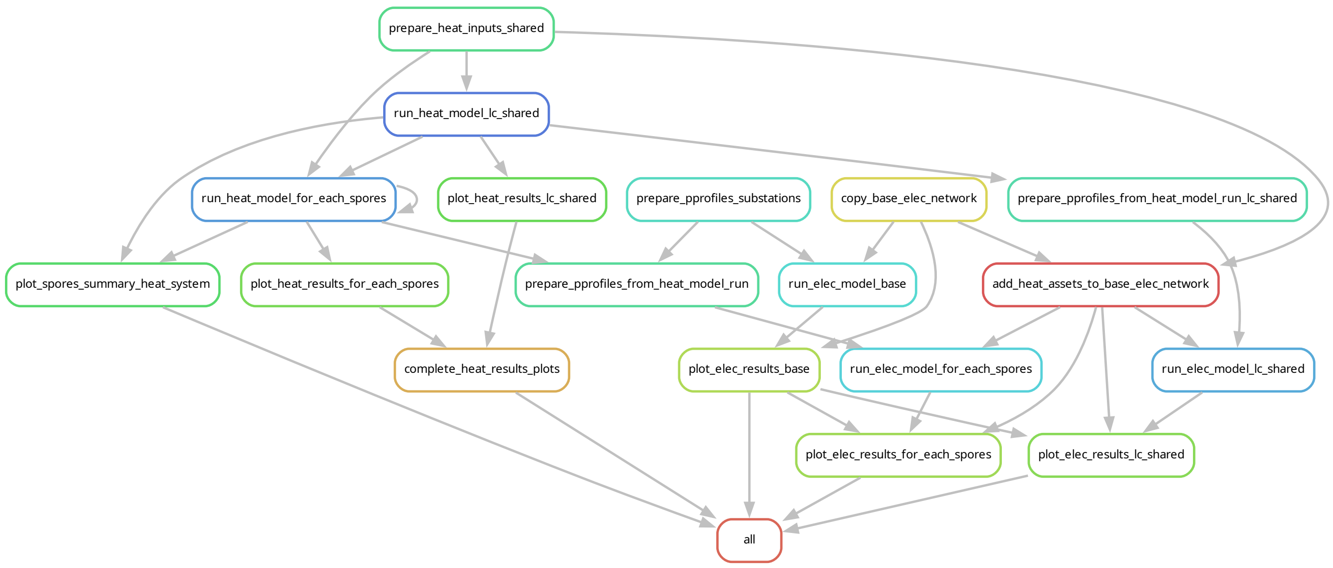

Figure S41. Directed acyclic graph of the model workflow for each scenario

Directed acyclic graph of jobs executed for each scenario, illustrating the simplified high-level workflow from raw data processing to generation of SPORES, running the DHN model for each SPORE, executing the power flow simulations, and quantifying electricity network impacts. The loop rule `run_heat_model_for_each_spores` corresponds to the generation of 515 SPORES per scenario, through intensification and diversification of DHN technology deployment.

Table S1. District heating technology parameters

Technology	Parameter	Value	Unit
Central air-source heat pump	investment	2,039,000	€/MW_th
	COP	temperature-dependent	–
	lifetime	15	years
Central CHP (green gas)	investment cost	2,000,000	€/MW_th
	heat efficiency	0.45	–
	electric efficiency	0.45	–
	lifetime	15	years
Central CHP (hydrogen)	investment cost	2,100,000	€/MW_th
	heat efficiency	0.45	–
	electric efficiency	0.45	–
	lifetime	15	years
Central electric boiler	investment cost	305,000	€/MW_th
	efficiency	0.9	–
	lifetime	15	years
Central gas boiler (green gas)	investment cost	200,000	€/MW_th

Continued on next page

Technology	Parameter	Value	Unit
	efficiency	0.8	–
	lifetime	15	years
Central gas boiler (hydrogen)	investment cost	270,000	€/MW_th
	efficiency	0.7	–
	lifetime	15	years
Central (deep) geothermal heat	investment cost	2,500,000	€/MW_th
	lifetime	15	years
	Seasonal performance factor	6	–
Central solar thermal heat	investment cost	435,000	€/MW_th
	lifetime	15	years
High-temperature residual heat	investment cost	1,200,000	€/MW_th
	lifetime	15	years
Central short-term storage (TES)	investment cost	250,000	€/MW
	energy-to-power ratio	6	hours
	charge efficiency	0.99	–
	discharge efficiency	0.99	–
	lifetime	30	years
Seasonal storage (HT-ATES)	investment cost	199,550	€/MW
	charge efficiency	1	–
	discharge efficiency	1	–
	round-trip efficiency	0.7	–
	full load hours	3,000	hours
	Seasonal performance factor	50	–
	lifetime	30	years
Heat distribution pipeline (high-temperature network)	investment cost	100	€/MW/m
	maximum capacity	250	MW
	losses	0.00001	%/m
	lifetime	30	years
Waste incinerator (waste-to-energy)	investment cost	600,000	€/MW
	efficiency	0.80	–
	lifetime	15	years
